# Optimal effectiveness of heart failure management — an umbrella review of meta-analyses examining the effectiveness of interventions to reduce (re)hospitalizations in heart failure

**DOI:** 10.1007/s10741-021-10212-8

**Published:** 2022-03-03

**Authors:** Frederique J. Hafkamp, Rene A. Tio, Luuk C. Otterspoor, Tineke de Greef, Gijs J. van Steenbergen, Arjen R. T. van de Ven, Geert Smits, Hans Post, Dennis van Veghel

**Affiliations:** 1 Netherlands Heart Network, Veldhoven, The Netherlands; 2 Catharina Hospital, Eindhoven, The Netherlands; 3 St. Anna Hospital, Geldrop, The Netherlands; 4 Primary care group Pozob, Veldhoven, The Netherlands

**Keywords:** Heart failure related hospitalizations, Interventions, Medication, Invasive therapy, Rehabilitations, Care pathways

## Abstract

Heart failure (HF) is a major health concern, which accounts for 1–2% of all hospital admissions. Nevertheless, there remains a knowledge gap concerning which interventions contribute to effective prevention of HF (re)hospitalization. Therefore, this umbrella review aims to systematically review meta-analyses that examined the effectiveness of interventions in reducing HF-related (re)hospitalization in HFrEF patients. An electronic literature search was performed in PubMed, Web of Science, PsycInfo, Cochrane Reviews, CINAHL, and Medline to identify eligible studies published in the English language in the past 10 years. Primarily, to synthesize the meta-analyzed data, a best-evidence synthesis was used in which meta-analyses were classified based on level of validity. Secondarily, all unique RCTS were extracted from the meta-analyses and examined. A total of 44 meta-analyses were included which encompassed 186 unique RCTs. Strong or moderate evidence suggested that catheter ablation, cardiac resynchronization therapy, cardiac rehabilitation, telemonitoring, and RAAS inhibitors could reduce (re)hospitalization. Additionally, limited evidence suggested that multidisciplinary clinic or self-management promotion programs, beta-blockers, statins, and mitral valve therapy could reduce HF hospitalization. No, or conflicting evidence was found for the effects of cell therapy or anticoagulation. This umbrella review highlights different levels of evidence regarding the effectiveness of several interventions in reducing HF-related (re)hospitalization in HFrEF patients. It could guide future guideline development in optimizing care pathways for heart failure patients.

## Introduction

Heart failure (HF) is a major health concern, with mortality ranging from 5 to 40% [1], corresponding with a fivefold increased risk of death, compared to the general population [2]. It is even estimated that HF patients have a worse life expectancy than the majority of cancer patients, with a median survival of approximately 2 to 3 years [3, 4]. More than 400,000 patients in the USA are being diagnosed with HF, annually [5]. Moreover, prevalence rates are progressively rising and are expected to increase with 46% from 2012 to 2030 [6, 7].

In addition, heart failure is the diagnosis with the highest readmission rates among all diseases [8–11], as it accounts for 1 to 2% of all hospital admissions [12, 13]. In elderly people, it is the major cause of hospitalization [8]. Most patients are hospitalized at least once a year after diagnosis (i.e., 68 to 78% of patients) [8, 14, 15], and more than one-fourth is at risk of being readmitted within 30 days after initial diagnosis [8, 15–18]. Comparatively to prevalence rates, the total number of hospitalizations is also expected to rise, by 50% in the near future [19, 20].

Hospitalization places a great burden on patients [21]. Patients may experience various limitations in their activities of daily living [22–24], which highly impact their quality of life and level of satisfaction [21, 25]. Moreover, aside from a reduced quality of life, patients who are hospitalized have a significantly higher risk of death than non-hospitalized patients [26, 27]. Additionally, hospitalization due to HF places a great burden on the healthcare system, as it accounts for more than half of total healthcare costs [28, 29] corresponding with more than > 15 billion dollars a year for the American healthcare system [24, 30, 31]. HF is the most costly condition in western countries and since long time hospitalization for HF even exceeds the hospitalization costs for both cancer and myocardial infarction combined [32, 33]. Accordingly, hospitalization is judged as a highly important outcome measure in (inter)national literature and registries [34, 35].

Nevertheless, despite the rising prevalence rates, it seems that up to 40% of hospitalizations could be classified as preventable [36–40]. Therefore, the reduction of hospitalizations is the most promising factor as target to improve patients’ reported experiences or outcomes and to reduce the costs of HF management [25, 41, 42]. The combined measure of patient outcomes and costs are the main goal in value based healthcare, a well-known and promising strategy in healthcare in order to improve patient value [43–45].

Multiple previous studies examined the effect of various interventions to reduce (re)hospitalization in HF, mostly in patients with an left ventricular ejection fraction (LVEF) < 40% (i.e., patients with HFrEF) [46], but contrasting findings are found within the literature regarding the effectiveness of these interventions in reducing hospital admissions [47, 48]. Moreover, there is some considerable heterogeneity in strategies and methods used in previous studies [49]. Some studies, for example, focused on remote monitoring to prevent readmissions, while others examined quality improvement of interventions or transitional care systems [36, 37, 50–52]. Therefore, there remains a gap in information concerning which interventions could effectively contribute to effective prevention of HF hospitalization or readmission [47, 48, 53, 54].

Hence, even though multiple interventions have been included in the guidelines for treatment of HF [46, 55], there is a compelling need of a comprehensive overview of which types of interventions prove effective specifically in *reducing HF hospitalizations*, especially in HFrEF patients. This umbrella review therefore aims to systematically review all published meta-analyses conducted in the past 10 years that examined the incremental effect of different interventions in addition to standard care, to reduce (re)hospitalization in HFrEF patients, in order to highlight different levels of evidence regarding their effectiveness.

## Methods

The systematic review protocol of this review was registered, in accordance with the PRISMA guidelines, at the International Prospective Register of Systematic Reviews (PROSPERO) on July 6, 2020 (registration number: 247872).

### Search strategy

An electronic literature search was performed in PubMed, Web of Science, PsycInfo, Cochrane Reviews, CINAHL, and Medline to identify eligible studies published in the English language from January 2010 up to the end of June 2020. Search terms were developed using MeSH terms. Key words were related to (1) interventions, (2) heart failure, (3) hospitalization, and (4) meta-analysis (Table 1).

**Table 1:** Search strategy for each database

**Database**	**Search terms**
PubMed	(((((((((((((((((((((((((((((((((((((((((((((((((((((((((((((((((((((((((((((((((((((((((((((((((((((((((((((((((((((((telemedicine[Title/Abstract]) OR (telecare[Title/Abstract])) OR (teleconsultation[Title/Abstract])) OR (telecommunication[Title/Abstract])) OR (home monitoring[Title/Abstract])) OR (monitoring[Title/Abstract])) OR (tele*[Title/Abstract])) OR (tele med*[Title/Abstract])) OR (tele-med*[Title/Abstract])) OR (telehealth[Title/Abstract])) OR (tele-health[Title/Abstract])) OR (remote consult*[Title/Abstract])) OR (remote monitoring[Title/Abstract])) OR (remote patient monitoring[Title/Abstract])) OR (structured telephone support[Title/Abstract])) OR (structured scheduled telephone support[Title/Abstract])) OR (telephone support[Title/Abstract])) OR (telecardiol*[Title/Abstract])) OR (home care services[Title/Abstract])) OR (disease management[Title/Abstract])) OR (patient care team[Title/Abstract])) OR (patient discharge[Title/Abstract])) OR (patient education[Title/Abstract])) OR (patient aftercare[Title/Abstract])) OR (patient care planning[Title/Abstract])) OR (home care services[Title/Abstract])) OR (manage*[Title/Abstract])) OR (comprehensive discharge planning[Title/Abstract])) OR (discharge planning[Title/Abstract])) OR (hospital discharge[Title/Abstract])) OR (patient care planning[Title/Abstract])) OR (multidisciplinary care[Title/Abstract])) OR (care management[Title/Abstract])) OR (transition*[Title/Abstract])) OR (comprehensive health care[Title/Abstract])) OR (process of care[Title/Abstract])) OR (comprehensive care[Title/Abstract])) OR (multidisciplinary care[Title/Abstract])) OR (improve*[Title/Abstract])) OR (promot*[Title/Abstract])) OR (enhanc*[Title/Abstract])) OR (optimi*[Title/Abstract])) OR (quality of health care[Title/Abstract])) OR (improvement initiative[Title/Abstract])) OR (process* improvement[Title/Abstract])) OR (management quality circles[Title/Abstract])) OR (total quality management[Title/Abstract])) OR (guideline adherence[Title/Abstract])) OR (clinical competence[Title/Abstract])) OR (rehabilitation centers[Title/Abstract])) OR (exercise therapy[Title/Abstract])) OR (rehabilitation[Title/Abstract])) OR (sports[Title/Abstract])) OR (physicial exertion[Title/Abstract])) OR (exertion[Title/Abstract])) OR (exercise[Title/Abstract])) OR (rehabilit*[Title/Abstract])) OR (lifestyle intervent*[Title/Abstract])) OR (life-style intervent*[Title/Abstract])) OR (psychotherapy[Title/Abstract])) OR (psychotherap*[Title/Abstract])) OR (psycholog*[Title/Abstract])) OR (psycholog* intervent*[Title/Abstract])) OR (self-care[Title/Abstract])) OR (relaxation therapy[Title/Abstract])) OR (counseling[Title/Abstract])) OR (cognitive therapy[Title/Abstract])) OR (behaviour therapy[Title/Abstract])) OR (behavior therapy[Title/Abstract])) OR (meditation[Title/Abstract])) OR (hypnotherap*[Title/Abstract])) OR (psycho-educat*[Title/Abstract])) OR (psychoeducat*[Title/Abstract])) OR (motiv* intervent*[Title/Abstract])) OR (health education[Title/Abstract])) OR (self-management[Title/Abstract])) OR (action plan*[Title/Abstract])) OR (medication[Title/Abstract])) OR (medication* treatment[Title/Abstract])) OR (pharmacotherapy[Title/Abstract])) OR (device* implantation[Title/Abstract])) OR (medication adherence[Title/Abstract])) OR (patient compliance[Title/Abstract])) OR (adherent[Title/Abstract])) OR (non-compliant[Title/Abstract])) OR (noncompliance[Title/Abstract])) OR (nonadherent[Title/Abstract])) OR (nonadherence[Title/Abstract])) OR (prescription drug[Title/Abstract])) OR (dosage forms[Title/Abstract])) OR (prescribed[Title/Abstract])) OR (pill*[Title/Abstract])) OR (invasisve HF monitoring[Title/Abstract])) OR (implanted monitoring devices[Title/Abstract])) OR (CRT[Title/Abstract])) OR (biventricular pacing[Title/Abstract])) OR (drug therapy[Title/Abstract])) OR (intervention[Title/Abstract])) OR (interven*[Title/Abstract]))OR (immunization[Title/Abstract]))) OR (e-health[Title/Abstract])) OR (program[Title/Abstract])) OR (mobile health[Title/Abstract])) OR (mhealth[Title/Abstract])) OR (after-hours care[Title/Abstract])) OR (integrated delivery of health care[Title/Abstract])) OR (managed care programs[Title/Abstract])) OR (technological interventions[Title/Abstract])) OR (inventions[Title/Abstract])) OR (automation[Title/Abstract])) OR (program evaluation[Title/Abstract])) OR (standard of care[Title/Abstract])) AND (((((heart failure[Title/Abstract]) OR (cardiac failure[Title/Abstract])) OR (congestive*[Title/Abstract])) OR (left ventricular dysfunction[Title/Abstract])) OR (CHF[Title/Abstract]))) AND ((((((readmission*hospitalization*[Title/Abstract]) OR (rehospitalization*[Title/Abstract])) OR (admission*[Title/Abstract])) OR (re-admission*[Title/Abstract])) OR (readmission*[Title/Abstract])) OR (length of stay[Title/Abstract]))) AND (((((meta analysis[Title/Abstract]) OR (meta-analysis[Title/Abstract])) OR (meta analy*[Title/Abstract])) OR (metaanaly*[Title/Abstract])) OR (meta-analy*[Title/Abstract]))
Cochrane library	#1(Telemedicine):ti,ab,kw OR (telecare):ti,ab,kw OR (teleconsultation):ti,ab,kw OR (telecommunication):ti,ab,kw OR (home monitoring):ti,ab,kw OR (monitoring):ti,ab,kw OR (tele*):ti,ab,kw OR (tele med):ti,ab,kw OR (tele-med*):ti,ab,kw OR (telehealth*):ti,ab,kw OR (tele-health*):ti,ab,kw OR (remote consult*):ti,ab,kw OR (remote monitoring):ti,ab,kw OR (remote patient monitoring):ti,ab,kw OR (structured telephone support):ti,ab,kw OR (structured scheduled telephone support):ti,ab,kw OR (telephone support):ti,ab,kw OR (telecardiol*):ti,ab,kw OR (home care services):ti,ab,kw OR (disease management):ti,ab,kw OR (patient care team):ti,ab,kw OR (patient discharge):ti,ab,kw OR (patient education):ti,ab,kw OR (patient aftercare):ti,ab,kw OR (patient care planning):ti,ab,kw OR (home care services):ti,ab,kw OR (manage*):ti,ab,kw OR (comprehensive discharge planning):ti,ab,kw OR (discharge planning):ti,ab,kw OR (hospital discharge):ti,ab,kw OR (patient care planning):ti,ab,kw OR (multidisciplinary care):ti,ab,kw OR (care management):ti,ab,kw OR (transition*):ti,ab,kw OR (comprehensive health care):ti,ab,kw OR (process of care):ti,ab,kw OR (comprehensive care):ti,ab,kw OR (multidisciplinary care):ti,ab,kw OR (improve*):ti,ab,kw OR (promot*):ti,ab,kw OR (enhanc*):ti,ab,kw OR (optimi*):ti,ab,kw OR (quality of health care):ti,ab,kw OR (improvement initiative):ti,ab,kw OR (process* improvement):ti,ab,kw OR (management quality circles):ti,ab,kw OR (total quality management):ti,ab,kw OR (guideline adherence):ti,ab,kw OR (clinical competence):ti,ab,kw OR (*rehabilitation centers):ti,ab,kw OR (exercise therapy):ti,ab,kw OR (*rehabilitation):ti,ab,kw OR (sports):ti,ab,kw OR (physical exertion):ti,ab,kw OR (exertion):ti,ab,kw OR (exercise):ti,ab,kw OR (rehabilit*):ti,ab,kw OR (lifestyle intervent*):ti,ab,kw OR (life-style intervent*):ti,ab,kw OR (psychotherapy):ti,ab,kw OR (psychotherap*):ti,ab,kw OR (psycholog*):ti,ab,kw OR (psycholog* intervent*):ti,ab,kw OR (self-care):ti,ab,kw OR (relaxation therapy):ti,ab,kw OR (counseling):ti,ab,kw OR (cognitive therapy):ti,ab,kw OR (behaviour therapy):ti,ab,kw OR (behavior therapy):ti,ab,kw OR (meditation):ti,ab,kw OR (hypnotherap*):ti,ab,kw OR (psycho-educat*):ti,ab,kw OR (psychoeducat*):ti,ab,kw OR (motiv* intervent*):ti,ab,kw OR (health education):ti,ab,kw OR ( self-management):ti,ab,kw OR (action plan*):ti,ab,kw OR (Medication):ti,ab,kw OR (medication* treatment):ti,ab,kw OR (pharmacotherapy):ti,ab,kw OR (device* implantation):ti,ab,kw OR (medication adherence):ti,ab,kw OR (patient compliance):ti,ab,kw OR (adherent):ti,ab,kw OR (non-compliant):ti,ab,kw OR (noncompliance):ti,ab,kw OR (nonadherent):ti,ab,kw OR (nonadherence):ti,ab,kw OR (prescription drugs):ti,ab,kw OR (dosage forms):ti,ab,kw OR (prescribed):ti,ab,kw OR (pill* ORinvasive HF monitoring):ti,ab,kw OR (implanted monitoring devices):ti,ab,kw OR (CRT):ti,ab,kw OR (biventricular pacing):ti,ab,kw OR (drug therapy):ti,ab,kw OR (intervention):ti,ab,kw OR (interven*):ti,ab,kw OR (e-health):ti,ab,kw OR (program):ti,ab,kw OR (mobile health):ti,ab,kw OR (mhealth):ti,ab,kw OR (after-hours care):ti,ab,kw OR (integrated delivery of health care):ti,ab,kw OR (managed care programs):ti,ab,kw OR (technological interventions):ti,ab,kw OR (inventions):ti,ab,kw OR (automation):ti,ab,kw OR (program evaluation):ti,ab,kw OR (standard of care):ti,ab,kw OR (OR influenza):ti,ab,kw #2(meta analysis):ti,ab,kw OR (meta-analysis):ti,ab,kw OR (meta analy*):ti,ab,kw OR (metaanaly*):ti,ab,kw OR (meta-analy*):ti,ab,kw #3(hospitalization*):ti,ab,kw OR (rehospitalization*):ti,ab,kw OR (admission*):ti,ab,kw OR (re-admission*):ti,ab,kw OR (readmission*):ti,ab,kw OR (length of stay):ti,ab,kw #4(*Heart failure):ti,ab,kw OR (cardiac failure):ti,ab,kw OR (congestive*):ti,ab,kw OR (left ventricular dysfunction):ti,ab,kw OR (CHF):ti,ab,kw #5#1 AND #2 AND #3 AND #4
Web of Science	#1: TS = (Telemedicine OR telecare OR teleconsultation OR telecommunication OR home monitoring OR monitoring OR tele* OR tele med OR telemed* OR telehealth* OR telehealth* OR remote consult* OR remote monitoring OR remote patient monitoring OR structured telephone support OR structured scheduled telephone support OR telephone support OR telecardiol* OR home care services OR disease management OR patient care team OR patient discharge OR patient education OR patient aftercare OR patient care planning OR home care services OR manage* OR comprehensive discharge planning OR discharge planning OR hospital discharge OR patient care planning OR multidisciplinary care OR care management OR transition* OR comprehensive health care OR process of care OR comprehensive care OR multidisciplinary care OR improve* OR promot* OR enhanc* OR optimi* OR quality of health care OR improvement initiative OR process* improvement OR management quality circles OR total quality management OR guideline adherence OR clinical competence OR *rehabilitation centers OR exercise therapy OR *rehabilitation OR sports OR physical exertion OR exertion OR exercise OR rehabilit* OR lifestyle intervent* OR life-style intervent* OR psychotherapy OR psychotherap* OR psycholog* OR psycholog* intervent* OR self-care OR relaxation therapy OR counseling OR cognitive therapy OR behaviour therapy OR behavior therapy OR meditation OR hypnotherap* OR psycho-educat* OR psychoeducat* OR motiv* intervent* OR health education OR self-management OR action plan* OR Medication OR medication* treatment OR pharmacotherapy OR device* implantation OR medication adherence OR patient compliance OR adherent OR non-compliant OR noncompliance OR nonadherent OR nonadherence OR prescription drugs OR dosage forms OR prescribed OR pill* ORinvasive HF monitoring OR implanted monitoring devices OR CRT OR biventricular pacing OR drug therapy OR intervention OR interven* OR e-health OR program OR mobile health OR mhealth OR after-hours care OR integrated delivery of health care OR managed care programs OR technological interventions OR inventions OR automation OR program evaluation OR standard of care) #2: TS = (meta analysis OR meta-analysis OR meta analy* OR metaanaly* OR meta-analy*) #3: TS = (hospitalization* OR rehospitalization* OR admission* OR re-admission* OR readmission* OR length of stay) #4: TS = (*Heart failure OR cardiac failure OR congestive* OR left ventricular dysfunction OR CHF) #5: #4 AND #3 AND #2 AND #1
Psycinfo	TX ( Telemedicine OR telecare OR teleconsultation OR telecommunication OR home monitoring OR monitoring OR tele* OR tele med OR tele-med* OR telehealth* OR tele-health* OR remote consult* OR remote monitoring OR remote patient monitoring OR structured telephone support OR structured scheduled telephone support OR telephone support OR telecardiol* OR home care services OR disease management OR patient care team OR patient discharge OR patient education OR patient aftercare OR patient care planning OR home care services OR manage* OR comprehensive discharge planning OR discharge planning OR hospital discharge OR patient care planning OR multidisciplinary care OR care management OR transition* OR comprehensive health care OR process of care OR comprehensive care OR multidisciplinary care OR improve* OR promot* OR enhanc* OR optimi* OR quality of health care OR improvement initiative OR process* improvement OR management quality circles OR total quality management OR guideline adherence OR clinical competence OR *rehabilitation centers OR exercise therapy OR *rehabilitation OR sports OR physical exertion OR exertion OR exercise OR rehabilit* OR lifestyle intervent* OR life-style intervent* OR psychotherapy OR psychotherap* OR psycholog* OR psycholog* intervent* OR self-care OR relaxation therapy OR counseling OR cognitive therapy OR behaviour therapy OR behavior therapy OR meditation OR hypnotherap* OR psycho-educat* OR psychoeducat* OR motiv* intervent* OR health education OR self-management OR action plan* OR Medication OR medication* treatment OR pharmacotherapy OR device* implantation OR medication adherence OR patient compliance OR adherent OR non-compliant OR noncompliance OR nonadherent OR nonadherence OR prescription drugs OR dosage forms OR prescribed OR pill* ORinvasive HF monitoring OR implanted monitoring devices OR CRT OR biventricular pacing OR drug therapy OR intervention OR interven* OR e-health OR program OR mobile health OR mhealth OR after-hours care OR integrated delivery of health care OR managed care programs OR technological interventions OR inventions OR automation OR program evaluation OR standard of care) AND TX ( meta analysis OR meta-analysis OR meta analy* OR metaanaly* OR meta-analy*) AND TX ( hospitalization* OR rehospitalization* OR admission* OR re-admission* OR readmission* OR length of stay) AND TX ( *Heart failure OR cardiac failure
Medline	AB ( Telemedicine OR telecare OR teleconsultation OR telecommunication OR home monitoring OR monitoring OR tele* OR tele med OR tele-med* OR telehealth* OR tele-health* OR remote consult* OR remote monitoring OR remote patient monitoring OR structured telephone support OR structured scheduled telephone support OR telephone support OR telecardiol* OR home care services OR disease management OR patient care team OR patient discharge OR patient education OR patient aftercare OR patient care planning OR home care services OR manage* OR comprehensive discharge planning OR discharge planning OR hospital discharge OR patient care planning OR multidisciplinary care OR care management OR transition* OR comprehensive health care OR process of care OR comprehensive care OR multidisciplinary care OR improve* OR promot* OR enhanc* OR optimi* OR quality of health care OR improvement initiative OR process* improvement OR management quality circles OR total quality management OR guideline adherence OR clinical competence OR *rehabilitation centers OR exercise therapy OR *rehabilitation OR sports OR physical exertion OR exertion OR exercise OR rehabilit* OR lifestyle intervent* OR life-style intervent* OR psychotherapy OR psychotherap* OR psycholog* OR psycholog* intervent* OR self-care OR relaxation therapy OR counseling OR cognitive therapy OR behaviour therapy OR behavior therapy OR meditation OR hypnotherap* OR psycho-educat* OR psychoeducat* OR motiv* intervent* OR health education OR self-management OR action plan* OR Medication OR medication* treatment OR pharmacotherapy OR device* implantation OR medication adherence OR patient compliance OR adherent OR non-compliant OR noncompliance OR nonadherent OR nonadherence OR prescription drugs OR dosage forms OR prescribed OR pill* ORinvasive HF monitoring OR implanted monitoring devices OR CRT OR biventricular pacing OR drug therapy OR intervention OR interven* OR e-health OR program OR mobile health OR mhealth OR after-hours care OR integrated delivery of health care OR managed care programs OR technological interventions OR inventions OR automation OR program evaluation OR standard of care OR) AND AB ( meta analysis OR meta-analysis OR meta analy* OR metaanaly* OR meta-analy) AND AB ( hospitalization* OR rehospitalization* OR admission* OR re-admission* OR readmission* OR length of stay) AND AB ( Heart failure OR cardiac failure OR congestive* OR left ventricular dysfunction OR CHF)
CINAHL	AB ( Telemedicine OR telecare OR teleconsultation OR telecommunication OR home monitoring OR monitoring OR tele* OR tele med OR tele-med* OR telehealth* OR tele-health* OR remote consult* OR remote monitoring OR remote patient monitoring OR structured telephone support OR structured scheduled telephone support OR telephone support OR telecardiol* OR home care services OR disease management OR patient care team OR patient discharge OR patient education OR patient aftercare OR patient care planning OR home care services OR manage* OR comprehensive discharge planning OR discharge planning OR hospital discharge OR patient care planning OR multidisciplinary care OR care management OR transition* OR comprehensive health care OR process of care OR comprehensive care OR multidisciplinary care OR improve* OR promot* OR enhanc* OR optimi* OR quality of health care OR improvement initiative OR process* improvement OR management quality circles OR total quality management OR guideline adherence OR clinical competence OR *rehabilitation centers OR exercise therapy OR *rehabilitation OR sports OR physical exertion OR exertion OR exercise OR rehabilit* OR lifestyle intervent* OR life-style intervent* OR psychotherapy OR psychotherap* OR psycholog* OR psycholog* intervent* OR self-care OR relaxation therapy OR counseling OR cognitive therapy OR behaviour therapy OR behavior therapy OR meditation OR hypnotherap* OR psycho-educat* OR psychoeducat* OR motiv* intervent* OR health education OR self-management OR action plan* OR Medication OR medication* treatment OR pharmacotherapy OR device* implantation OR medication adherence OR patient compliance OR adherent OR non-compliant OR noncompliance OR nonadherent OR nonadherence OR prescription drugs OR dosage forms OR prescribed OR pill* ORinvasive HF monitoring OR implanted monitoring devices OR CRT OR biventricular pacing OR drug therapy OR intervention OR interven* OR e-health OR program OR mobile health OR mhealth OR after-hours care OR integrated delivery of health care OR managed care programs OR technological interventions OR inventions OR automation OR program evaluation OR standard of care) AND AB ( meta analysis OR meta-analysis OR meta analy* OR metaanaly* OR meta-analy*) AND AB ( hospitalization* OR rehospitalization* OR admission* OR re-admission* OR readmission* OR length of stay) AND AB ( Heart failure OR cardiac failure OR congestive* OR left ventricular dysfunction OR CHF)

Ample differences existed in the classification of categories of interventions depicted in the existing literature. For example, previous reviews classified interventions in either educational interventions, pharmacological interventions, telemonitoring (TM), structured telephone support (STS), nurse home visits, nurse care management, and disease management clinics [41]; or discharge planning protocols, comprehensive geriatric assessments, discharge support arrangements, and educational interventions [56]; or case management interventions, clinical interventions, and multidisciplinary interventions [53]; or predischarge interventions, postdischarge interventions, and interventions bridging the transition [57]. A list of 4 categories of interventions was derived following a scoping review that combine the most common interventions aimed at reducing hospital (re)admissions, cardiac rehabilitation, care pathways, medication, and invasive treatment. Both general terms linked to the concept of interventions (e.g., programs, inventions, therapy) and terms for specific examples of (categories of) interventions were included in the search strategy.

### Eligibility criteria

Search results of all databases were combined, and duplicates were removed. Titles and abstracts were screened against the following inclusion criteria: (1) a meta-analysis was conducted, on (2) randomized controlled trials (RCTs), (3) that examined the effectiveness of (3.a) cardiac rehabilitation, or (3.b) care pathways, or (3.c) medication, or (3.d) invasive therapy, (4) in patients with an established diagnosis of chronic heart failure, (5) with an LVEF < 40, (6) with a primary or secondary objective to evaluate the effect on reduction of (7) HF-related hospitalization or readmissions, (8) as compared to usual care, (9) conducted in the past 10 years, (10) followed patients for at least three months, and (11) were reported in English. Meta-analyses that included both RCTs and observational or cohort studies were not excluded. Yet only the included RCTs (and corresponding meta-analyzed effect sized) were extracted and used for our analyses. Only meta-analyses that reported at least one meta-analyzed effect estimate for HF-related admissions were included. In order to assure objective assessment, the title and abstract screening were independently conducted by two researchers (FH, TG). In case of disagreement between reviewers, points of disagreement were discussed in order to reach consensus. For full-text screening, inter-rate reliability was calculated using Cohen’s kappa.

Studies were excluded when the patient population was not primarily diagnosed with heart failure (e.g., patients with diabetes and comorbid heart failure). Additionally, if studies examined HF patients in combination with other patient groups yet did not report data on the individual patient groups, the study was excluded, as we would otherwise be unable to make a distinction between the differences in patient groups. Furthermore, studies that only reported data on a combined endpoint (e.g., mortality in conjunction with HF-hospitalization) and meta-analyses that examined risk stratification, prognostic factors, or lifestyle advice in patients were excluded. Moreover, meta-analyses were also excluded when examining a specific subgroup of HF patients (e.g., patients with and LVAD) or when examining a broader category of patients that could possibly include HF patients (e.g., “older patients” in general).

### Quality assessment

The “A MeaSurement Tool to Assess systematic Reviews 2” (AMSTAR 2) was used to assess the methodological quality of included meta-analyses [58]. AMSTAR 2 consists of 16 items, of which 10 items were retained from the original AMSTAR tool. Response options for the items were “yes,” “partial yes,” and “no,” with “yes” responses denoting a positive result. The overall score of this tool was converted to high quality, moderate quality, low quality, and critically low quality. High quality was achieved when studies possessed no or one non-critical weakness; moderate quality was achieved when studies had more than one non-critical weakness; low quality was achieved when studies had one critical flaw, with or without a non-critical weakness; and critically low quality was achieved when studies exhibited more than one critical flaw with or without non-critical weaknesses. Critical domains are depicted in Table 2 [58]. In order to assure objective assessment, the quality assessment was independently conducted by two researchers (GS, TG). In case of disagreement between reviewers, points of disagreement were discussed in order to reach consensus (RT).

**Table 2:** Critical domains of the AMSTAR 2

Registered protocol before commencement of the review
Risk of bias from individual studies being included in the review
Appropriateness of meta-analytical methods
Consideration of risk of bias when interpreting the results of the review
Assessment of presence and likely impact of publication bias

### Data extraction

A standardized extraction form was used to extract data from the included studies. Sociodemographic data (e.g., age, sex), number of participants, left ventricular ejection fraction, type of intervention and control, follow-up period, effect size, and conclusion were extracted from either the individual RCT or the meta-analysis in which the RCT was included. Only the most recent meta-analysis was included when multiple articles were written by the same authors on the same dataset. Comparisons were made between the different categories of interventions in terms of effectiveness in reducing HF-related (re)hospitalization. Interventions were classified as having a significant effect on HF-related (re)hospitalization (as compared to usual care) based on their own reported RR statistics, findings, and conclusions.

### Data synthesis

Interventions were first classified into the four predefined categories (i.e., cardiac rehabilitation, care pathways, medication, and invasive therapy) and subsequently divided into more detailed classes of interventions (e.g., TM and STS) to examine the exact effect of all unique interventions.

#### Primary analysis: meta-analyses

To synthesize the data, a best-evidence synthesis was used as primary analysis, in which meta-analyses were classified based on level of internal and external validity [59]. The levels of evidence regarding the significance or non-significance of a relationship between the intervention and HF-related hospitalization among studies were ranked according to the following statements: (1) strong evidence: consistent findings (> 75% of the studies reported consistent findings) in multiple high quality studies; (2) moderate evidence: consistent findings (> 75% of the studies reported consistent findings) in one high-quality study and two or more moderate quality studies or in three or more weak quality studies; (3) limited evidence: generally consistent findings (> 75% of the studies reported consistent findings) in a high quality study or in two or fewer moderate quality studies; (4) no evidence: no studies could be found; (5) conflicting evidence: conflicting findings.

#### Secondary analysis: extracted RCTs

It was expected that multiple meta-analyses would report identical RCTs, as it was previously found that the amount of redundancy and duplication among reviews is substantial [60, 61]. Therefore, the corrected covered area (CCA) was calculated, which is a measure of duplicates in meta-analyses divided by the frequency of duplicates, reduced by the number of original publications $(\text{Corrected}\;\mathrm{covered}\;\mathrm{area}=\frac{N-r}{r\;c-r})$ [62]. A CCA of 0–5% is considered as slight overlap, while 6–10%, 11–15%, > 15% are respectively regarded as moderate, high, and very high overlap. In order to prevent bias as a result of duplicated data, a secondary analysis was conducted to control for the effects of overlap. All unique RCTs were extracted from the meta-analyses. Individual risk ratios (RRs) and 95% CIs for each intervention were calculated using Review Manager V.5.4. or extracted from the meta-analyses. The *I*^2^-statistic was used to present the heterogeneity of intervention effect. When the *I*^2^-statistic was statistically significant, a random-effects model was used in analyses. The RR-statistics found in our own meta-analyses were compared to the reported effects in the published meta-analyses.

## Results

### Search results

**Fig. 1:**
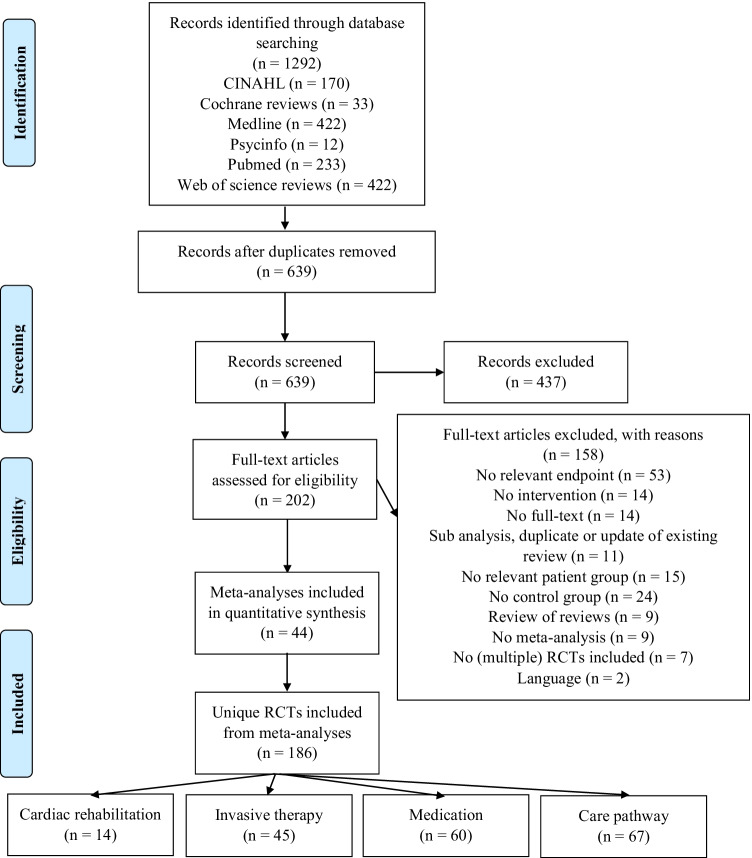
Flow diagram of study inclusion. RCT: randomized controlled trial

After removal of duplicate meta-analyses, 639 titles and abstracts were screened (see Fig. 1). A total of 202 full-text articles were assessed for eligibility, of which 44 were included in our analyses. Cohen’s kappa for full-text screening was 0.76, indicating substantial agreement [63]. Median year of publication of all included meta-analyses was 2018. The 44 included meta-analyses encompassed 348 RCTs of which 186 were unique RCTs regarding interventions to prevent HF hospitalization (Table 3). Of these 186 unique RCTs, 44 were classified as invasive therapy, 14 as cardiac rehabilitation, 60 as medication, and 67 as care pathways (Table 4). The CCA for cardiac revalidation was $\frac{(19-14)}{\left(\left(14\times 2\right)-14\right)}= \frac{5}{14}=36\%$, the CCA for invasive therapy was $\frac{(86-45)}{\left(\left(45\times 15\right)-45\right)}= \frac{41}{630}=7\%$, the CCA for medication was $\frac{(100-60)}{\left(\left(64 \times 14\right)-60\right)}= \frac{40}{836}=5\%$, and the CCA for care pathways was $\frac{(138-67)}{\left(\left(67 \times 15\right)- 67\right)}= \frac{73}{896}=8\%$. This indicates a moderate to very high overlap in included RCTs [62].

**Table 3:** Overlap between different meta-analyses in included RCTs

		1	2	3	4	5	6	7	8	9	10	11	12	13	14	15	16	17	18	19	20	21	22
Abraham et al. [87]	2002			x																			
Abraham et al. [88]	2004																						
Adamson et al. [89]	2003	x																					
Adamson et al. [90]	2011	x			x													x					
Al-khatib et al. [91]	2010				x																		
Angermann et al. [92]	2012																				x		
Antonicelli et al. [93]	2008																						
Asgar et al. [94]	2017						x																
Assmus et al. [95]	2006														x								
Assmus et al. [96]	2013														x	x							
Atienza et al. [97]	2004																						
Austin et al. [98]	2005								x														
Australia/New Zealand Heart Failure Group [99]	1997																						
Bartunek et al. [100]	2013														x								
Belardinelli et al. [101]	1999								x														
Belardinelli et al. [102]	2012								x														
Beller et al. [103]	1995																						
Bentkover et al. [104]	2007																						
Beta-Blocker evaluation of survival trial [105]	2001																						
Biannic et al. [106]	2012																				x		
Bielecka-Dabrowa et al. [107]	2009									x													
Blue et al. [108]	2001																						
Boccanelli et al. [109]	2009											x								x			
Böhm et al. [110]	2016																	x					
Bolli et al. [111]	2011															x							
Boriani et al. [112]	2017				x													x					
Boyne et al. [113]	2012																						
Bristow et al. [114]	1996																						
Brown et al. [115]	1995																						
Lok et al. [116]	2007																x						
Capomolla et al. [117]	2002																x						
Cazeau et al. [118]	2001			x																			
CDMR [119]	1988																						
Chan et al. [120]	2007											x								x			
Chaudhry et al. [69]	2010																				x		
Chen et al. [121]	2018																						
Chung [122]	2021																						
CIBIS [123]	1994																						
CIBIS-II [124]	1999																						
Cicoira et al. [125]	2002											x											
Cleland et al. [126]	2004																						
Cline et al. [127]	1998																x						
Cohn and Tognoni [128]	2001													x									
Cokkinos et al. [129]	2006																						
Colucci et al. [130]	1996																						
Consensus et al. [241]	2000																						
Cowie et al. [131]	2014																						
Dalal et al. [132]	2019								x														
Dar et al. [133]	2009																						
Dargie [134]	2001																						
Daubert et al. [135]	2009																						
Dendale et al. [136]	2012																				x		
Dewalt et al. [137]	2012																						
Di Biase et al. [138]	2016		x			x																	
DIG [139]	1997																						
Domenichini et al. [140]	2016				x													x					
Domingo et al. [141]	2011																						
Domingues et al. [142]	2011																						
Doughty et al. [143]	2002																x						
Ducharme et al. [144]	2005																x						
Dunagan et al. [145]	2005																						
Ekman et al. [146]	1998																x						
Ellingsen et al. [147]	2017								x														
Erhardt et al. [148]	1995																						
Fisher et al. [149]	1994																						
Fox et al. [150]	2008																		x				
Fragasso et al. [151]	2006																						
Gallagher et al. [152]	2017																						
Gasparini et al. [153]	2006			x																			
Gattis et al. [154]	1999																				x	x	
Giannini et al. [155]	2016						x																
Giannuzzi et al. [156]	2003								x														
Giordano et al. [157]	2009																				x	x	
Goldberg et al. [158]	2003																						
Goldstein et al. [159]	1999																						
Granger et al. [160]	2000																						
Granger et al. [161]	2003													x									
Hamaad et al. [162]	2005									x													
Hambrecht et al. [163]	1995																						
Hambrecht et al. [164]	2000								x														
Hamshere et al. [165]	2015														x								
Hanconk et al. [166]	2012												x										
Hansen et al. [167]	2018				x																		
Heldman et al. [168]	2014														x	x							
Heldman et al. [168]	2014															x							
Higgins et al. [169]	2003			x																			
Hindricks et al. [170]	2014				x													x					
Idris et al. [171]	2015																						
Jaarsma et al. [47]	2008																x						
Jolly et al. [172]	2009								x														
Jones and Wong [173]	2013		x																				
Kashem et al. [174]	2008																						
Kasper et al. [175]	2002																x						
Koehler et al. [176]	2011										x										x		
Komajda [177]	2004																						
Kraai et al. [178]	2016																						
Krum et al. [179]	2013																				x		
Krumholz et al. [180]	2002																						
Landolina et al. [181]	2012				x													x					
Laramee et al. [182]	2003																				x	x	
Linde et al. [183]	2002			x																			
Leclercq et al. [184]	2007			x																			
Linde et al. [185]	2008			x																			
Liu et al. [186]	2012																x						
Lüthje et al. [187]	2015				x													x					
Luttik et al. [188]	2014																x						
Lyngå et al. [68]	2012																						
MacDonald et al. [189]	2011		x			x																	
Maggioni et al. [190]	2002																						
Margulies et al. [191]	2016																						
Marrouche et al. [192]	2018		x			x																	
Martinelli et al. [193]	2010			x																			
Mathiasen et al. [194]	2015														x								
Menasché [195]	2008															x							
Mcdonald et al. [196]	2002																						
McMurray et al. [197]	2003													x									
MERIT-HF [198]	1999																						
Morgan et al. [199]	2017				x																		
Mortara et al. [200]	2009																				x	x	
Moss et al. [201]	2002																						
Moss et al. [202]	2009			x																			
Mozid et al. [203]	2014														x								
Mueller et al. [204]	2007								x														
Node et al. [205]	2003									x													
Obadia et al. [206]	2018						x	x															
Packer et al. [207]	1993																						
Packer et al. [208]	1996																						
Packer et al. [209]	1996																						
Packer et al. [210]	2001																						
Passino et al. [211]	2006																						
Patel et al. [212]	2015														x								
Pätilä et al. [213]	2014														x	x							
Perin et al. [214]	2012														x								
Peters-klimm et al. [215]	2010																						
Pfeffer et al. [216]	1992																						
Piepoli et al. [217]	2008			x																			
Pinter et al. [218]	2009																						
Pitt et al. [219]	1999											x											
Pitt et al. [220]	2003											x											x
Pokushalov et al. [221]	2010																						
Pokushalov et al. [222]	2011																						
Prabhu et al. [223]	2017					x																	
Ramachandran et al. [224]	2007																				x	x	
Rosano et al. [225]	2003																						
Ruschitzka et al. [226]	2013																						
Sardu et al. [227]	2016																						
Scherr et al. [228]	2009																						
Schou et al. [229]	2013																x						
Sisk et al. [230]	2006												x								x	x	
Smith et al. [231]	2014																x						
Sola et al. [232]	2006									x													
Yusuf et al. [233]	1991													x									
Yusuf et al. [234]	1992													x									
Spargias et al. [235]	1999													x									
Stone et al. [236]	2018						x	x															
Sturm et al. [237]	2000																						
Swedberg et al. [238]	2010																		x				
Takano et al. [239]	2013									x													
Tang et al. [240]	2010			x																			
Consensus et al. [241]	2000																						
Thibault et al. [242]	2011			x																			
Thibault et al. [243]	2013																						
Tsuyuki et al. [244]	2004																				x	x	
Tuunanen et al. [245]	2008																						
Udelson et al. [246]	2010																			x			
Uretsky et al. [247]	1993																						
van Veldhuisen et al. [248]	2009																						
van Veldhuisen et al. [249]	2011				x													x					
Villani et al. [250]	2007																						
Villani et al. [251]	2014										x												
Vitale et al. [252]	2004																						
Vizzardi et al. [253]	2010											x											
Vrtovec et al. [254]	2008									x													
Vuorinen et al. [255]	2014																				x		
Weintraub et al. [256]	2010																						
Wierzchowiecki et al. [257]	2006																						
Willenheimer et al. [258]	2001																						
Wojnicz et al. [259]	2006									x													
Xie et al. [260]	2010									x													
Yamada et al. [261]	2007									x													
Young et al. [262]	2003				x																		
Zan [263]	2020										x												
Zannad et al. [264]	2011											x		x						x			x
Zannad et al. [265]	2018																						

		23	24	25	26	27	28	29	30	31	32	33	34	35	36	37	38	39	40	41	42	43	44
Abraham et al. [87]	2002		x												x								
Abraham et al. [88]	2004																x						
Adamson et al. [89]	2003																						
Adamson et al. [90]	2011																						
Al-khatib et al. [91]	2010																						
Angermann et al. [92]	2012									x									x				
Antonicelli et al. [93]	2008									x						x							
Asgar et al. [94]	2017																						
Assmus et al. [95]	2006																						
Assmus et al. [96]	2013																						
Atienza et al. [97]	2004	x												x									
Austin et al. [98]	2005																						
Australia/New Zealand Heart Failure Group [99]	1997														x								
Bartunek et al. [100]	2013																						
Belardinelli et al. [101]	1999												x										
Belardinelli et al. [102]	2012																						
Beller et al. [103]	1995				x																		
Bentkover et al. [104]	2007		x																				
Beta-Blocker evaluation of survival trial [105]	2001														x								
Biannic et al. [106]	2012																						
Bielecka-Dabrowa et al. [107]	2009																						
Blue et al. [108]	2001	x		x										x		x				x			
Boccanelli et al. [109]	2009				x	x																	
Böhm et al. [110]	2016																						
Bolli et al. [111]	2011																						
Boriani et al. [112]	2017																						
Boyne et al. [113]	2012															x							
Bristow et al. [114]	1996				x																		
Brown et al. [115]	1995				x																		
Lok et al. [116]	2007	x												x									
Capomolla et al. [117]	2002									x				x		x				x			
Cazeau et al. [118]	2001		x																				
CDMR [119]	1988														x								
Chan et al. [120]	2007					x																	
Chaudhry et al. [69]	2010									x						x							
Chen et al. [121]	2018												x										
Chung [122]	2021		x																				
CIBIS [123]	1994				x										x								
CIBIS-II [124]	1999														x								
Cicoira et al. [125]	2002					x																	
Cleland et al. [126]	2004											x											
Cline et al. [127]	1998																						
Cohn and Tognoni [128]	2001				x																		
Cokkinos et al. [129]	2006											x											
Colucci et al. [130]	1996				x																		
Consensus et al. [241]	2000				x																		
Cowie et al. [131]	2014												x										
Dalal et al. [132]	2019												x										
Dar et al. [133]	2009			x						x						x				x			
Dargie [134]	2001														x								
Daubert et al. [135]	2009																x						
Dendale et al. [136]	2012									x						x			x				
Dewalt et al. [137]	2012	x																					
Di Biase et al. [138]	2016						x	x															
DIG [139]	1997														x								
Domenichini et al. [140]	2016																						
Domingo et al. [141]	2011															x							
Domingues et al. [142]	2011									x													
Doughty et al. [143]	2002													x									
Ducharme et al. [144]	2005																						
Dunagan et al. [145]	2005			x												x				x			
Ekman et al. [146]	1998													x									
Ellingsen et al. [147]	2017																						
Erhardt et al. [148]	1995				x																		
Fisher et al. [149]	1994														x								
Fox et al. [150]	2008														x								
Fragasso et al. [151]	2006																				x		x
Gallagher et al. [152]	2017															x							
Gasparini et al. [153]	2006																						
Gattis et al. [154]	1999																						
Giannini et al. [155]	2016																						
Giannuzzi et al. [156]	2003												x										
Giordano et al. [157]	2009																		x	x			
Goldberg et al. [158]	2003									x						x							
Goldstein et al. [159]	1999				x																		
Granger et al. [160]	2000				x																		
Granger et al. [161]	2003														x								
Hamaad et al. [162]	2005																						
Hambrecht et al. [163]	1995												x										
Hambrecht et al. [164]	2000																						
Hamshere et al. [165]	2015																						
Hanconk et al. [166]	2012																						
Hansen et al. [167]	2018																						
Heldman et al. [168]	2014																						
Heldman et al. [168]	2014																						
Higgins et al. [169]	2003		x														x						
Hindricks et al. [170]	2014																						
Idris et al. [171]	2015															x							
Jaarsma et al. [47]	2008	x																					
Jolly et al. [172]	2009												x							x			
Jones and Wong [173]	2013						x	x	x									x					
Kashem et al. [174]	2008			x												x				x			
Kasper et al. [175]	2002			x										x		x			x	x			
Koehler et al. [176]	2011															x				x			
Komajda et al. [177]	2004				x																		
Kraai et al. [178]	2016															x							
Krum et al. [179]	2013															x							
Krumholz et al. [180]	2002			x												x				x			
Landolina et al. [181]	2012																						
Laramee et al. [182]	2003			x						x						x				x			
Linde et al. [183]	2002																						
Leclercq et al. [184]	2007																						
Linde et al. [185]	2008		x												x								
Liu et al. [186]	2012																						
Lüthje et al. [187]	2015																						
Luttik et al. [188]	2014																						
Lyngå et al. [68]	2012															x				x			
MacDonald et al. [189]	2011						x	x	x									x					
Maggioni et al. [190]	2002														x								
Margulies et al. [191]	2016																					x	
Marrouche et al. [192]	2018						x	x	x									x					
Martinelli et al. [193]	2010																						
Mathiasen et al. [194]	2015																						
Menasché [195]	2008																						
Mcdonald et al. [196]	2002															x							
McMurray et al. [197]	2003				x																		
MERIT-HF [198]	1999				x										x								
Morgan et al. [199]	2017																						
Mortara et al. [200]	2009			x												x				x			
Moss et al. [201]	2002														x								
Moss et al. [202]	2009		x												x		x						
Mozid et al. [203]	2014																						
Mueller et al. [204]	2007												x										
Node et al. [205]	2003																						
Obadia et al. [206]	2018																						
Packer et al. [207]	1993														x								
Packer et al. [208]	1996														x								
Packer et al. [209]	1996														x								
Packer et al. [210]	2001														x								
Passino et al. [211]	2006												x										
Patel et al. [212]	2015																						
Pätilä et al. [213]	2014																						
Perin et al. [214]	2012																						
Peters-klimm et al. [215]	2010															x							
Pfeffer et al. [216]	1992														x								
Piepoli et al. [217]	2008		x																				
Pinter et al. [218]	2009		x																				
Pitt et al. [219]	1999					x									x								
Pitt et al. [220]	2003														x								
Pokushalov et al. [221]	2010		x																				
Pokushalov et al. [222]	2011		x																				
Prabhu et al. [223]	2017						x	x	x									x					
Ramachandran et al. [224]	2007			x																			
Rosano et al. [225]	2003																				x		x
Ruschitzka et al. [226]	2013		x																				
Sardu et al. [227]	2016															x							
Scherr et al. [228]	2009															x				x			
Schou et al. [229]	2013																						
Sisk et al. [230]	2006			x																			
Smith et al. [231]	2014																						
Sola et al. [232]	2006																						
Yusuf et al. [233]	1991				x										x								
Yusuf et al. [234]	1992				x										x								
Spargias et al. [235]	1999																						
Stone et al. [236]	2018																						
Sturm et al. [237]	2000				x																		
Swedberg et al. [238]	2010				x										x								
Takano et al. [239]	2013																						
Tang et al. [240]	2010		x												x		x						
Consensus et al. [241]	2000				x																		
Thibault et al. [242]	2011																x						
Thibault et al. [243]	2013		x																				
Tsuyuki et al. [244]	2004	x								x													
Tuunanen et al. [245]	2008																				x		x
Udelson et al. [246]	2010																						
Uretsky et al. [247]	1993														x								
van Veldhuisen et al. [248]	2009														x								
van Veldhuisen et al. [249]	2011																						
Villani et al. [250]	2007																			x			
Villani et al. [251]	2014																						
Vitale et al. [252]	2004																				x		x
Vizzardi et al. [253]	2010					x																	
Vrtovec et al. [254]	2008																						
Vuorinen et al. [255]	2014															x							
Weintraub et al. [256]	2010																			x			
Wierzchowiecki et al. [257]	2006													x									
Willenheimer et al. [258]	2001												x										
Wojnicz et al. [259]	2006																						
Xie et al. [260]	2010																						
Yamada et al. [261]	2007																						
Young et al. [262]	2003																						
Zan [263]	2020																						
Zannad et al. [264]	2011				x	x									x								
Zannad et al. [265]	2018											x											

1, Adamson et al. [266]; 2, Agasthi et al. [267]; 3, Al-Majed et al. [268]; 4, Alotaibi et al. [269]; 5, AlTurki et al. [270]; 6, Benito-González et al. [271]; 7, Bertaina et al. [272]; 8, Bjarnason-Wehrens et al. [273]; 9, Bonsu et al. [274]; 10, Carbo et al. [275]; 11, de Vecchis et al. [276]; 12, Driscoll et al. [277]; 13, Emdin et al. [278]; 14, Fisher et al. [279]; 15, Fisher et al. [280]; 16, Gandhi et al. [281]; 17, Halawa et al. [282]; 18, Hartmann et al. [283]; 19, Hu et al. [284]; 20, Inglis et al. [285]; 21, Inglis et al. [286]; 22, Japp et al. [287]; 23, Jonkman et al. [288]; 24, Kang et al. [289]; 25, Klersy et al. [290]; 26, Komajda et al. [291]; 27, Le et al. [292]; 28, Ma et al. [293]; 29, Malik et al. [294]; 30, Moschonas et al. [295]; 31, Pandor et al. [296]; 32, Shah et al. [297]; 33, Sulaica et al. [298]; 34, Taylor et al. [299]; 35, Thomas et al. [300]; 36, Thomsen et al. [301]; 37, Tse et al. [302]; 38, Tu et al. [303]; 39, Turagam et al. [304]; 40, Uminski et al. [305]; 41, Xiang et al. [306]; 42, Zhang et al. [307]; 43, Zhang et al. [308]; 44, Zhou and Chen [309]

**Table 4:** Baseline characteristics of RCTs

**Included RCTs**	***N* (intervention)**	***N* (control)**	**Total**	**Mean age**	**% Male**	**Mean LVEF**	**Intervention**	**Control**	**Follow-up period**
Beller et al. [103]	130	63	193	61	76	28	Initial oral dose of 5 mg of *lisinopril*. The dose of diuretic therapy was adjusted based on the clinical condition of the patient, particularly to control edema	Matching placebo	3 months
Brown et al. [115]	116	125	241	62	82	25	The 24-week double-blind treatment period beginning with 10 mg of *fosinopril*. In the ensuing 3 weeks, patients were titrated to 20 mg of study medication (level TI), as tolerated	Matching placebo	N/R
CDMR [119]	200	100	300	57	83	25	*Captopril* (25 to 50 mg, three times a day)	Placebo	6 months
Consensus et al. [241]	127	126	263	71	56	< 40	*Enalapril* (2.5 to 40 mg/day)	Placebo	12 months
Erhardt et al. [148]	155	153	308	64	76	27	*Fosinopril* 10 mg	Matching placebo	12 weeks
Pfeffer et al. [216]	1115	1116	2231	60	83	31	*Captopril*	Placebo	36 months
Yusuf et al. [233]	1285	1284	2569	61	81	25	*Enalapril*	Placebo	41 months
Yusuf et al. [234]	2111	2117	4228	59	89	28	*Enalapril*	Placebo	42 months
Cleland et al. [126]	89	190	279	63	74	< 35	*Warfarin* with INR of 2.5	Aspirin or no antithrombotic therapy	27 months
Cokkinos et al. [129]	92	105	197	59	85	28	*Warfarin* was supplied as 5-mg tablets. The daily dose was 2.5–10 mg, with a target INR of 2–3	Placebo	19.5 months
Zannad et al. [265]	2507	2515	5022	66	77	34	*Rivaroxaban* 2.5 mg twice daily	Placebo	21 months
Cohn and Toghoni [128]	2511	2499	5010	63	80	27	*Valsartan* was initiated at a dose of 40 mg twice daily, and the dose was doubled every 2 weeks until a target dose of 160 mg twice daily was reached	Placebo	23 months
Granger et al. [160]	179	91	270	66	25	26	*Candesartan*, 4 mg, 8 mg and 16 mg	Matching placebo	12 months
Granger et al. [161]	1013	1015	2028	66	68	30	*Candesartan*, 4 mg, 8 mg, 16 mg, 32 mg	Matching placebo	34 months
Maggioni et al. [190]	185	181	67	63	71	28	*Valsartan*	Placebo	12 months
McMurray et al. [197]	N/R	N/R	7599	67	40	54	*Candesartan*	Matching placebo	N/R
Spargias et al. [235]	1734	243	1977	67	74	40	*Ramipril*	Placebo	N/R
Sturm [237]	51	49	100	52	90	17	*Atenolol*	Placebo	24 months
Australia/New Zealand Heart Failure Research Collaborative Group [99]	208	207	415	67	80	29	*Carvedilol*	Matching placebo	19 months
Beta-Blocker evaluation of survival trial [105]	1354	1354	2708	60	79	23	Initial oral dose of 3 mg of *bucindolol*, which was repeated twice daily for 1 week	Placebo	24 months
Bristow et al. [114]	261	84	345	60	78	23	Low-dose *Carvedilol* (6.25 mg BID), medium-dose *Carvedilol* (12.5 mg BID), and high-dose *Carvedilol* (25 mg BID)	Placebo	6 months
CIBIS [123]	320	321	641	N/R	N/R	25	2.5 mg *Bisoprolol*	2,5 mg placebo	1.9 years
CIBIS-II [124]	N/R	N/R	N/R	N/R	N/R	28	*Bisoprolol* 1.25 mg	Placebo	1.3 years
Colucci et al. [130]	232	134	366	55	86	23	*Carvedilol*	Placebo	213 days
Dargie [134]	975	984	1959	63	74	33	*Carvedilol*	Identical looking placebo	1.3 years
Fisher et al. [149]	25	25	50	63	100	22	*Metoprolol*, from 6.25 to 12.5 mg twice a day to 12.5 mg three times a day to 25 mg twice a day	Placebo	6 months
Goldstein et al. [159]	40	20	60	N/R	N/R	27	The initial dose of approximately 12.5 mg *Metoprolol* (one half of a 25 mg tablet) was administered once daily. The dose of metoprolol was increased to 25 mg and subsequently increased in steps of 50 mg to 100 mg and finally to 150 mg once daily	Matching placebo	26 weeks
Komajda [177]	N/R	N/R	572	N/R	N/R	< 40	*Enalapril*	Matching placebo	N/R
Merit-HF [198]	1990	2001	3991	64	78	28	*Metoprolol*	Placebo	1 year
Packer et al. [208]	133	145	278	61	73	22	*Carvedilol*, 25–50 mg BID	Placebo	6 months
Packer et al. [209]	696	398	1094	58	77	23	*Carvedilol*	Placebo	6.5 months
Packer et al. [210]	1156	1133	2289	63	80	20	*Carvedilol*	Placebo	10.4 months
van Veldhuisen et al. [248]	678	681	1359	76	70	29	*Nebivolol*	Placebo	21 months
Di Biase [138]	102	101	203	74	60	29	PVI + LAPWI + SVCI + CFAE	AMIO therapy	24 months
Jones and Wong [173]	26	26	52	63	87	22	PVI + linear then CFAEs	Rate control	12 months
MacDonald et al. [189]	22	19	41	63	78	20	PVI ± linear lesions + CFAEs	Rate control	6 months
Marrouche et al. [192]	179	184	363	85	61	32	PVI + / − additional lesions at discretion of operator	Rate and/or rhythm control	38 months
Prabhu et al. [223]	33	33	66	91	61	35	PVI + LAPWI	Rate control	6 months
DIG [139]	3397	3403	6800	64	78	29	*Digoxin*	Placebo	37 months
Packer et al. [207]	85	93	178	61	76	28	*Digoxin*	Placebo	3 months
Uretsky et al. [247]	42	46	88	64	90	29	*Digoxin*	Withdrawal of digoxin	3 months
Assmus et al. [95]	24	23	47	61	100	39–41	Intracoronary infusion of BMC or CPC	No cell infusion	3 months
Assmus et al. [96]	64	39	103	65	90	32–37	Intracoronary infusion of BMCs	Cell-free medium (placebo)	45.7 months
Bartunek et al. [100]	32	15	47	59	91	28	Patients in the cell therapy arm received bone marrow–derived cardiopoietic stem cells meeting quality release criteria	Standard of care comprising a beta-blocker, an angiotensin-converting enzyme inhibitor or angiotensin receptor blocker, and a diuretic with dosing and schedule tailored for maximal benefit and tolerability in accordance with practice guidelines for heart failure management	2 years
Bolli et al. [111]	16	7	23	57	100	30	Autologous CSCs were isolated from the right atrial appendage and re-infused intracoronarily 4 ± 1 months after surgery;	No treatment	12 months
Hamshere et al. [165]	15	15	30	56	86	42	G-CSF + BMSC	Peripheral placebo (saline)	12 months
Heldman et al. [168]	22	11	33	60	95	38–40	Mesenchymal stem cell group or bone marrow mononuclear cell group	Placebo	12 months
Heldman et al. [168]	38	21	59	61	100	36	Mesenchymal stem cell group or bone marrow mononuclear cell group	Placebo	12 months
Mathiasen et al. [194]	40	20	60	66	90	28	BMSC	Placebo	6 months
Menasché [195]	63	34	97	61	100	29	Cell suspension	Placebo solution consisting of the suspension medium without skeletal myoblasts	72 months
Mozid et al. [203]	14	2	16	70	94	31	G-CSF + BMSC	Placebo	6 months
Patel et al. [212]	24	6	30	59	100	26	BMAC infusion	Standard heart failure care	12 months
Pätilä et al. [213]	20	19	39	65	95	37	Injections of BMMC or vehicle intra-operatively into the myocardial infarction border area	Controls received only vehicle medium by syringes	12 months
Perin et al. [214]	20	10	30	61	80	39	Transendocardial delivery of ABMMNCs	Placebo	6 months
Austin et al. [98]	100	100	200	60	66	85% < 35	An 8-week cardiac rehabilitation program that was coordinated by the clinical nurse specialist. Patients attended classes twice weekly for a period of 2.5 h	Eight weekly monitoring of clinical status (functional performance, fluid status, cardiac rhythm, laboratory assessment) in the cardiology outpatients by the clinical nurse specialist	8 weeks
Belardinelli et al. [101]	50	49	99	59	88	28	The exercise group underwent exercise training for 14 months	The control group did not exercise	14 months
Belardinelli et al. [102]	63	60	123	59	78	37	The trained group underwent an ET program for 10 years. The training program consisted of 3 sessions per week at the hospital for 2 months, then 2 supervised sessions the rest of the year. Every 6 months, patients exercised at the hospital, and then they returned to a coronary club, where they exercised the rest of the year	The nontrained group was not provided with a formal ET program	120 months
Chen et al. [121]	19	18	27	61	36	36	Outpatient cardiac rehabilitation for 1 week, before starting home-based cardiac rehabilitation. Home-based cardiac rehabilitation was conducted by requesting the interventional group to carry out aerobic exercise at least 3 times per week, for a duration of at least 30 min each time	Instructed to maintain both their standard medical care and previous activity levels	3 months
Cowie et al. [131]	30	16	46	64	91		The hospital group attended a physiotherapist-led class	A DVD and booklet (replicating the class) was created for home use. Controls followed their usual HFNS care	5 years
Dalal et al. [132]	107	109	216	70	78	35	REACH-HF manual for patients with a choice of two structured exercise programs	No cardiac rehabilitation approach that included medical management according to national and local guidelines, including specialist heart failure nurse care	12 weeks
Ellingsen et al. [147]	78	81	261	60	81	29	HIIT and MCT had 3 supervised sessions per week on a treadmill or bicycle. HIIT included four 4-min intervals aiming at 90 to 95% of maximal heart rate separated by 3-min active recovery periods of moderate intensity. MCT sessions aimed at 60 to 70% of maximal heart rate	Patients were advised to exercise at home according to current recommendations and attended a session of moderate-intensity training at 50 to 70% of maximal heart rate every 3 weeks	3 months
Giannuzzi et al. [156]	45	45	90	60	N/R	25	The exercise protocol consisted of supervised continuous sessions of 30-min bicycle ergometry > 3 times a week (3 to 5 times) at 60% of the peak V˙ O_2_ achieved at the initial symptom-limited exercise testing. In addition to supervised sessions, patients were asked to take a brisk daily walk for > 30 min and intermittent unsupervised sessions of calisthenics (30 min) as part of the home-based exercise program	Educational support, but no formal exercise protocol	6 months
Hambrecht et al. [163]	12	10	22	52	27	26	Patients assigned to the training program remained in an intermediate care ward for the initial 3 weeks. Training sessions were conducted individually under strict supervision for the first 3 weeks. Patients exercised six times daily for 10 min on a bicycle ergometer	Patients assigned to the control group spent 3 days in an intermediate care ward for baseline evaluation. After discharge, medical therapy was continued, and patients were supervised by their private physicians	6 months
Hambrecht et al. [164]	36	37	73	54	100	27	2 weeks of in-hospital ergometer exercise for 10 min 4 to 6 times per day, followed by 6 months of home-based ergometer exercise training for 20 min per day at 70% of peak oxygen uptake	No intervention	6 months
Jolly et al. [172]	84	85	169	66	75	< 40	Three supervised exercise sessions to plan an individualized exercise program. These were followed by a home-based program, with home visits at 4, 10, and 20 weeks, telephone support at 6, 15, and 24 weeks, and a manual with details about safe progressive exercise and self-monitoring of frequency, duration, and intensity	Specialist heart failure nurse input in primary and secondary care through clinic and home visits that included the provision of information about heart failure, advice about self-management and monitoring of their condition, and titration of beta-blocker therapy	3 months
Mueller et al. [204]	25	25	50	55	100	< 40	Five indoor cycling sessions were performed weekly for a duration of 30 min, and all subjects walked outdoors for 45 min twice daily. Training duration was one month	Control subjects received usual clinical care, including verbal encouragement to remain physically active	1 month
Passino et al. [211]	44	41	85	N/R	N/R	35	The training group underwent a nine-month training program. The training program consisted of cycling on a bike for a minimum of 3 days per week, 30 min per day	Control patients continued their usual lifestyle	9 months
Willenheimer et al. [258]	27	27	54	N/R	N/R	35	Patients carried out cycle ergometer interval training at a heart rate corresponding to 80% of peak-VO2 ± 5 beats/min, for as long as possible during each interval	Control patients were asked not to change their degree of physical activity during the active study period	6 months
Abraham et al. [87]	228	225	453	64	68	22	Atrial-synchronized biventricular pacing	No pacing for six months, during which time medications for heart failure were to be kept constant	6 months
Abraham et al. [88]	101	85	186	64	89	25	Optimal medical treatment with active CRT and active ICD therapy	Optimal medical treatment and active ICD therapy	6 months
Bentkover et al. [104]	36	36	72	79	79	< 35	Biventricular pacing and ICD	ICD alone	6 months
Cazeau et al. [118]	29	29	58	63	75	23	Atriobiventricular (active) pacing	Ventricular inhibited (inactive) pacing	3 months
Chung [122]	9	9	18	76	76	30	A CRT–defibrillator device with LV coronary venous lead system	A dual-chamber ICD	12 months
Daubert et al. [135]	82	180	262	81	81	28	Patients who had undergone successful implantation were randomly assigned in a 2-to-1 scheme to a CRT ON group for 24 months	CRT OFF	24 months
Gasparini et al. [153]	33	36	69	67	94	26	BiV CRT	LV	12 months
Higgins et al. [169]	245	245	490	66	84	22	CRT-D	ICD	6 months
Linde et al. [183]	25	18	43	66	84	30	Biventricular VVIR pacing during two 3-month periods	Right-univentricular VVIR pacing during two 3-month periods	3 months
Leclercq et al. [184]	25	19	44	74	100	27	Biventricular VVIR pacing during two 3-month periods	Right-univentricular VVIR pacing during two 3-month periods	3 months
Linde et al. [185]	419	191	610	79	79	27	Active CRT	Control	12 months
Martinelli et al. [193]	27	27	54	59	68	30	Device was initially programmed to BiVP, crossed to RVP and crossed back to BiVP	Device was initially programmed to RVP, crossed to BiVP and crossed back to RVP	18 months
Moss et al. [201]	742	490	1232	65	85	23	ICD	Conventional medical therapy	20 months
Moss et al. [202]	1089	731	1820	75	75	24	Cardiac-resynchronization therapy with biventricular pacing	ICD alone	2.4 years
Piepoli et al. [217]	44	45	89	72	72	24	CRT-P/CRT-D	Medical	12 months
Pinter et al. [218]	36	36	72	79	79	23	CRT-D	ICD	6 months
Pokushalov et al. [221]	91	87	178	90	90	29	CRT-P + CABG	CABG	18 months
Pokushalov et al. [222]	13	13	26	96	96	27	BMMC + active CRT	BMMC + inactive CRT	6 months
Ruschitzka et al. [226]	404	405	809	72	72	27	CRT capability turned on	CRT capability turned off	19.4 months
Tang et al. [240]	894	904	1798	83	83	23	ICD + CRT	ICD alone	40 months
Thibault et al. [242]	60	61	121	75	75	24	biventricular CRT	LV CRT	6 months
Thibault et al. [243]	44	41	85	71	71	25	CRT-D	ICD	12 months
Young et al. [262]	182	187	369	68	78	24	Combined CRT and ICD capabilities	ICD activated, CRT off	6 months
Fragasso et al. [151]	34	31	65	65	96	35	*Trimetazidine*, 20 mg three times daily	Placebo	13 months
Rosano et al. [225]	16	16	32	66	75	33	20 mg t.d.s. *trimetazidine*	Placebo t.d.s	6 months
Tuunanen et al. [245]	12	7	19	58	79	34	*Trimetazidine*	Placebo	3 months
Vitale et al. [252]	23	24	47	78	85	29	*Trimetazidine*	Placebo	6 months
Margulies et al. [191]	154	146	300	62	69	25	*Liraglutide*	Placebo	6 months
Fox et al. [150]	5479	5438	10,917	60	83	32	*Ivabradine* 7.5 MG BID	Placebo	19 months
Swedberg et al. [238]	3241	3264	6505	65	76	29	*Ivabradine* 7.5 MG BID	Placebo	22.9 months
Asgar et al. [94]	50	42	92	75	77	38	Treated with the MitraClip	This retrospective comparator group consisted of medically managed patients	22–33 months
Giannini et al. [155]	60	60	120	76	70	34	MitraClip	Optimal medical therapy	17 months
Obadia et al. [206]	152	152	304	71	79	33	Percutaneous mitral-valve repair	medical therapy alone	12 months
Stone et al. [236]	302	312	614	73	67	31	Transcatheter mitral-valve repair plus medical therapy	Medical therapy alone	16.5 months
Boccanelli et al. [109]	188	193	381	63	84	40	*Canrenone*	Placebo	12 months
Chan et al. [120]	23	25	48	63	83	27	Candesartan 8 mg and *spironolactone* 25 mg once daily	Candesartan 8 mg and a matching identical placebo once daily	12 months
Cicoira et al. [125]	54	52	106	67	86	33	*Spironolactone* treatment, at an initial dose of 25 mg once daily	Placebo	12 months
Pitt et al. [219]	822	841	1663	65	73	25	*Spironolactone*, 25 mg	Matching placebo	24 months
Pitt et al. [220]	3319	3313	6632	64	71	33	*Eplerenone*	Placebo	16 months
Udelson et al. [246]	116	109	225	63	84	27	*Eplerenone*, 50 mg/d	Placebo	9 months
Vizzardi et al. [253]	65	65	130	65	N/R	36	25 mg of *spironolactone* once daily	Matching placebo	44 months
Zannad et al. [264]	1364	1373	2737	69	78	26	*Eplerenone* 50 mg/d	Placebo	21 months
Atienza et al. [97]	164	174	338	68	60	36	1 individual session prior to discharge by nurse, 1 visit to physician, 3-monthly follow-up visits and tele-monitoring	Usual care (discharge planning according to protocol)	509 days
Blue et al. [108]	84	81	165	75	48	Severe 40%	Planned home visits of decreasing frequency, supplemented by telephone contact as needed. The aim was to educate the patient about heart failure and its treatment, optimize treatment (drugs, diet, exercise), monitor electrolyte concentrations, teach self-monitoring and management, liaise with other health care and social workers as required, and provide psychological support	Patients in the usual care group were managed as usual by the admitting physician and, subsequently, general practitioner. They were not seen by the specialist nurses after hospital discharge	12 months
Lok et al. [116]	118	122	240	71	79	31	An intensive follow-up of the patients during 1 year at a HF outpatient clinic led by a HF physician and a cardiovascular nurse. Verbal and written comprehensive education was imparted about the disease and the aetiology, medication, compliance and possible adverse events. Patients were advised about individualized diet with salt and fluid restriction, weight control, early recognition of worsening HF, when to call a healthcare provider, and about physical exercise and rest. An appointment with a dietician was made. The nurse asked the patient about his or her social and medical circumstances and performed a short physical examination. The physician assessed the clinical condition of the patient, the laboratory results and ECG, performed a physical examination, and, together with the nurse, proposed a treatment regimen	Their routine care was no doubt largely according to the guideline of the European Society of Cardiology prevailing at that time (version 2001), with optimal application of medical therapy including the target dose or high dose of HF medication	12 months
Capomolla et al. [117]	112	122	234	56	84	31	The objectives of the multidisciplinary staff are prevention and functional recovery of consequences of acute hemodynamic instabilization	Patients were referred to their primary care physician and cardiologist. During follow-up the process of care was driven by the patient’s needs into a heterogeneous range of emergency room management, hospital admission, and outpatient access	12 months
Cline et al. [127]	80	110	190	76	55	36	The education program consisted of two 30-min information visits by a nurse during primary hospitalization and a 1-h information visit for patients and family 2 weeks after discharge	Routine clinical practice	1 year
Dendale et al. [136]	80	80	160	76	65	33	Patients were seen in the outpatient heart failure clinic with additional planned visits at 3 and 6 months. Daily patient telemonitoring was conducted with specified alert limits set for each patient. Alterations in patient status were forwarded to the general practitioner and heart failure clinic for subsequent patient follow-up and management	Usual care	6 months
Dewalt et al. [137]	303	302	605	61	52	< 40	The intervention began with a 1-h educational session with a clinical pharmacist or health educator during a regular clinic visit. Patients were given an educational booklet designed for low literacy patients and a digital scale. As part of the educational session, patients were taught to identify signs of heart failure exacerbation, perform daily weight assessment, and adjust their diuretic dose. The program coordinator then made scheduled follow-up phone calls and monthly during months	Patients enrolled in the control group received a general heart failure education pamphlet written at approximately the 7th grade level and continued with usual care from their primary physician	12 months
Doughty et al. [143]	100	97	197	74	56	34	One-on-one education with the study nurse was initiated at the first clinic visit. A patient diary, for daily weights, medication record, clinical notes and appointments, and education booklet were provided. Group education sessions (each lasting 1.5–2 h) were offered, two within 6 weeks of hospital discharge and a further after 6 months	Continued under the care of their GP with additional follow-up measures as usually recommended by the medical team responsible for their in-patient care	12 months
Ducharme et al. [144]	115	115	230	70	73	35	Patients in the intervention group were referred to a multidisciplinary specialized heart failure outpatient clinic where they were evaluated by the study team within 2 weeks of hospital discharge	Received treatment and appropriate follow-up according to the standards of the attending physicians but without further direct contact with the research team or the planned intervention	6 months
Ekman et al. [146]	79	79	158	N/R	N/R	43	The structured-care program was based on a nurse monitored, outpatient clinic, run in cooperation with the study doctors, who were responsible for optimal pharmacological treatment	Usual care	5 months
Gallagher et al. [152]	20	20	40	64	75	25	A licensed clinical social worker reviewed adherence data daily during the first 7 days after discharge and weekly thereafter and contacted participants who were nonadherent for two or more days per week. During these phone calls, the social worker inquired about consequences of nonadherence, and assessed and responded to reasons for missed doses	For participants assigned to passive monitoring, adherence data were recorded but not monitored by the study team	1 months
Hancock et al. [166]	16	12	28	85	44	43	An assessment visit by a consultant cardiologist who initiated a plan of treatment, followed by visits at one to two weekly intervals within the home by heart failure specialist nurses. The HFSNs enacted the plan, including blood tests, assessment of symptoms and signs, educational advice, and medication titration	Routine care	6 months
Jaarsma et al. [47]	340	339	679	72	66	34	(A) 2 individual session by cardiologist, 9 visits to nurse, possibility to contact nurse (B) 2 individual sessions by cardiologist, 18 visits to nurse, 2 home visits, 2 multidisciplinary sessions, follow-up telephone contact by nurse	Usual care (standard management by cardiologist)	18 months
Kasper et al. [175]	102	98	200	62	61	27	Patients received nurse-led care coordination linked to a multidisciplinary team composed of a heart failure nurse, cardiologist, and patient’s primary care physician. Patients were contacted via telephone at preplanned intervals after discharge, in addition to scheduled visits within the community	Patients received unrestricted follow-up care from their primary physicians, who received a baseline heart failure management plan, as documented in the patient's chart	6 months
Krumholz et al. [180]	44	44	88	74	57	38	The study intervention was based on five sequential care domains for chronic illness, including patient knowledge of the illness, the relation between medications and illness, the relation between health behaviors and illness, knowledge of early signs and symptoms of decompensation and where and when to obtain assistance	Patients assigned to the control group received all usual care treatments and services ordered by their physicians	12 months
Liu et al. [186]	53	53	200	63	66	29	The patient was cared for by an HF team consisting of 3 cardiologists specialized in HF care, one psychologist, one dietary assistant, and two case managers	The primary care physician was responsible for patient evaluation, treatment and clinic visits. Neither scheduled follow-up nor specialized HF nurses were available	6 months
Luttik et al. [188]	92	97	200	73	63	32	Follow-up by the HF clinic	Follow-up by their GP	12 months
Lyngå et al. [68]	166	153	344	73	75	57% < 30	Patients randomized to the IG were given an electronic scale to install in their homes	The patients in the CG were informed to contact the HF clinic on a special telephone in the case of a weight gain of .2 kg in 3 days	12 months
Mcdonald et al. [196]	51	47	98	71	66	37	Patients systematically received specialist nurse-led education and specialist dietitian consults on three or more occasions during the index admission. The education program focused on daily weight monitoring, disease and medication understanding, and salt restriction	Patients underwent investigations for HF, including echocardiography and right and left heart catheterization where indicated. Optimal medical therapy was administered	3 months
Schou et al. [229]	460	460	200	69	63	32	Patients allocated to an extended follow-up completed the following program: visits at 1–3-month intervals at the discretion of the investigators	Usual care by a GP	9 months
Smith et al. [231]	92	106	198	63	66	30	The intervention began with four weekly group visit appointments followed by a 5th “booster” appointment held 6 months after randomization	HF care from their existing treatment team both during and after hospitalization	12 months
Tsuyuki et al. [244]	140	136	276	74	55	31	The essential components of the patient support program were simplified into 5 basic areas: salt and fluid restriction, daily weighing, exercise alternating with rest periods, proper medication use, and knowing when to call their physician (early recognition of worsening symptoms)	Usual care	6 months
Wierzchowiecki et al. [257]	64	65	129	81	N/R	36	Multidisciplinary care	Routine care	6 months
Bielecka-Dabrowa et al. [107]	41	27	68	57	85	29	*Atorvastatin* 40 mg daily for 2 months (8 weeks) and next 10 mg for 4 months	DCM was treated according to present standards without statin therapy	6 months
Hamaad et al. [162]	12	9	23	67	86	32	*Atorvastatin*, 40 mg once daily	Placebo	32.8 months
Node et al. [205]	23	25	48	48	69	34	*Simvastatin*	Placebo	3.5 months
Sola et al. [232]	54	54	108	33	63	33	*Atorvastatin*	No statin treatment	12 months
Takano et al. [239]	288	286	577	63	N/R	34	*Pitavastatin*	Control	35.5 months
Vrtovec et al. [254]	55	55	110	62	61	25	*Atorvastatin* (10 mg/day)	No statins	12 months
Wojnicz et al. [259]	36	38	74	38	81	28	*Atorvastatin*	Placebo	6 months
Xie et al. [260]	N/R	N/R	81	N/R	N/R	38	*Atorvastatin* (10–20 mg/day)	Routine treatment	12 months
Yamada et al. [261]	19	19	38	64	79	34	*Atorvastatin* 10 mg/day	Conventional treatment	31 months
Angermann et al. [92]	352	363	715	69	71	30	Included the following elements: (1) in-hospital face-to-face contact between specialist nurse, patient, and relatives to explain the intervention, practice supervision of blood pressure, heart rate and symptoms; (2) telephone-based structured monitoring; (3) up titration of heart failure medication; (4) needs-adjusted specialist care, which nurses coordinated with patients’ physician(s); (5) measures for appropriate education and supervision of interveners to ensure high intervention quality	Standard postdischarge planning, which typically included treatment plans, comprehensive discharge letters, and fixed appointments with GPs or cardiologists within 7–14 days	6 months
Chaudhry et al. [69]	826	827	1653	61	52	71% < 40	Structured (daily) telephone-based monitoring (of symptoms and weight) via an interactive voice response system	Standard optimal care. Followed by local physician. Guideline based therapy	6 months
Domingues et al. [142]	48	63	111	63	68	29	Structured (weekly for 1st month, every 15 days for following 2 months) telephone-based education and monitoring signs and symptoms of decompensation	Usual care that consisted of the follow-up of the patient at the return appointment at the outpatient clinic without any telephone contact	3 months
Dunagan et al. [145]	76	75	151	70	44	75% < 40	The intervention group received additional education from study nurses during scheduled telephone contact	Educational packet describing the causes of HF, the basic principles of treatment, their role in routine care and monitoring of their condition, and appropriate strategies for managing a HF exacerbation	12 months
Gattis et al. [154]	90	91	181	67	68	30	Clinical pharmacist-led medication review and patient education. Regularly scheduled telephone contact (at 2, 12 and 24 weeks) to detect clinical deterioration early	Usual care	6 months
Krum et al. [179]	188	217	405	73	61	36	Nurse-led telephone monitoring. Participant responded to computer-generated CHF self-monitoring questions by pressing the numbers on the touch-phone keypad. Nurse survey incoming calls daily and responded to preset variations to participant's parameters	Usual care involved standard general practice management of heart failure	12 months
Laramee et al. [182]	141	146	287	71	54	< 40	Four major components were (1) early discharge planning and coordination of care, (2) individualized and comprehensive patient and family education, (3) 12 weeks of enhanced telephone follow-up and surveillance, and (4) promotion of optimal CHF medications and medication doses (ACEIs or ARBs and BBs)	Standard care, typical of a tertiary care hospital, and all conventional treatments requested by the attending physician	3 months
Mortara et al. [200]	301	160	461	60	85	29	The patients enrolled in HT strategies 2 and 3 transmitted weekly records of the following data to the coordinating center via an automated interactive voice response system: weight; heart rate; systolic arterial pressure; dyspnea score; asthenia score; oedema score; changes in therapy; and blood results	Patients allocated to the control arm were discharged as normal from the hospital	12 months
Peters-klimm et al. [215]	97	100	197	70	72	38	The design of the intervention addressed 4 elements: delivery system design, self-management support, decision support, clinical information systems	No case management was applied	12 months
Ramachandran et al. [224]	25	25	50	45	78	21	Intervention group participants were managed in the heart failure clinic and received disease, medication and self-management education and telephonic disease management which consisted of reinforcement of information and drug dose modification	Usual care in the heart failure clinic	6 months
Sisk et al. [230]	203	203	406	N/R	N/R	< 40	An in-person appointment was arranged for each intervention participant, which included symptom and disease education and referral to additional patient services (if required). Follow-up telephone calls consisted of participant assessment, recording of admission information reinforcement of self-monitoring and administration of a food-frequency questionnaire	Usual care patients received federal consumer guidelines for managing systolic dysfunction but no other intervention	12 months
Adamson et al. [89]	N/R	N/R	32	59	38	29	Permanent right-ventricular implantable hemodynamic monitor system similar to a single-lead pacemaker	Historical controls	17 months
Adamson et al. [90]	198	202	400	55	69	23	Expert disease management conforming to consensus recommendations coupled with hemodynamic information from the IHM	The control group received expert disease management with frequent and random nursing calls	12 months
Al-khatib et al. [91]	76	75	151	63	62	25	Remote monitoring of ICDs using the Medtronic CareLink transmission monitor	Quarterly ICD interrogations in clinic classified as standard of care	12 months
Antonicelli et al. [93]	28	29	57	78	61	35	Patients were contacted by telephone at least once a week by the team to obtain information on symptoms and adherence to prescribed treatment, as well as blood pressure, heart rate, bodyweight and 24-h urine output data for the previous day. A weekly ECG transmission was also required. Evaluation of these parameters was followed by reassessment of the therapeutic regimen and modification whenever needed	Standard care based on routinely scheduled clinic visits from a team specialized in CHF patient management	12 months
Biannic et al. [106]	35	38	73	78	79	32	TM during 3 months, after which participants all received usual care up until 1 year	Usual care	3 months
Böhm et al. [110]	497	505	1002	66	80	27	Telemedicine alerts enabled, triggered by intrathoracic fluid index threshold crossing, which was programmed at the investigator’s discretion. The fluid status monitoring algorithm detects changes in thoracic impedance resulting from accumulation of intrathoracic fluid as an early sign of developing cardiac decompensation	To not transmit alerts	23 months
Boriani et al. [112]	428	437	865	66	76	27	Received a monitor for scheduled remote device checks, and automatic alerts for lung fluid accumulation atrial tachyarrhythmia, and system integrity were enabled. In-office device checks were requested to re-arm alerts which had been temporarily inactivated due to previous transmissions	In-office follow-ups alone	24 months
Boyne et al. [113]	185	197	382	71	59	36	The patients in the intervention arm received a device, with a liquid crystal display and four keys, connected to a landline phone. Daily pre-set dialogues were communicated about symptoms, knowledge, and behaviour, being answered by touching one of the keys and sent to a server and to the nurses’ desktop	Nurse-led usual care was given according to the latest European Society of Cardiology guidelines, including oral and written educational information, and psychosocial support as needed	12 months
Capomolla et al. [310]	67	66	133	57	88	29	The objectives of the multidisciplinary staff are prevention and functional recovery of consequences of acute hemodynamic instabilization. The team members also have the task of creating, analyzing, and correcting the organization that supports the process of treatment identified in an individual care plan	Patients were referred to their primary care physician and cardiologist. During follow-up the process of care was driven by the patient’s needs into a heterogeneous range of emergency room management, hospital admission, and outpatient access	11 months
Dar et al. [133]	91	91	182	72	66	61% < 40	Home telemonitoring. Daily measurement, manual transmission of weight, blood pressure, heart rate, oxygen saturation and symptoms	Standard care	6 months
Domenichini et al. [140]	39	41	80	68	94	29	OptiVolw or CorVueTM functions and alarms activated	The OptiVolw or CorVueTM functions switched on, as Group 1, whereas the alarms were not activated	12 months
Domingo et al. [141]	44	48	92	66	71	36	Motiva System with educational videos, motivational messages	Patients were instructed to record their weight, blood pressure, and heart rate each morning before breakfast	12 months
Giordano et al. [157]	230	230	460	57	85	28	Patient telemonitoring involving medical and nursing professionals. Daily transmission of cardiac parameters was monitored by a cardiologist, general practitioner and nurse, who assessed the patient's clinical status, providing consultation or triage. Nurse-driven telephone contacts to assess patient status and treatment regimen adherence were conducted weekly, or biweekly, dependent on patient status	Referred to their primary care physician. A structured follow-up with the cardiologist at 12 months in the hospital outpatient department and the appointment with the primary care physician within 2 weeks from the discharge were planned	12 months
Goldberg et al. [158]	138	142	280	N/R	N/R	22	The system includes an electronic scale placed in patients’ homes. Patients were instructed to weigh themselves and respond to yes/no questions about heart failure related symptoms twice daily. The attending physician individualized the symptom questions and weight goals for each patient at the time of enrollment	Patients were instructed to contact their physician for weight increases of more than a prespecified amount or if their symptoms of heart failure worsened. These patients were asked to bring a copy of their home weight log to study visits	6 months
Hansen et al. [167]	102	108	210	63	83	28	Receive quarterly automated follow-up via telemetry	Receive quarterly personal contact with a physician	13 months
Hindricks et al. [170]	331	333	664	66	81	26	In the telemonitoring group, transmitted data were reviewed by study investigators according to their clinical routine. In parallel, transmitted data were reviewed by a central monitoring unit composed of trained study nurses and supporting physicians	In the control group, no study participant had access to telemonitoring data until study completion. All patients were treated according to European guidelines	12 months
Idris et al. [171]	14	14	28	63	39	23	Daily remote monitoring of blood pressure, heart rate, oxygen saturation, and weight via the telemonitoring system for 3 months	Standard care	3.6 months
Kashem et al. [174]	24	24	48	54	74	25	Blood pressure, pulse, steps/day, and weight together with symptoms were entered. The most recent laboratory data and medication were entered by the practice staff, and the patient was instructed to review medications and laboratory values and transmit any questions to the practice	Usual care	12 months
Koehler et al. [176]	354	356	710	67	82	27	The system is based on a wireless Bluetooth device, together with a personal digital assistant, as the central structural element. Data transfer was performed with the use of cell phone technologies. The patient performed a daily self-assessment and the data were transferred to the responsible telemedical center	Usual care	26 months
Koehler et al. [311]	N/R	N/R	710	67	81	< 30	The system is based on a wireless Bluetooth device together with a personal digital assistant as the central structural element. The patient performed a daily self-assessment and the data was transferred to the telemedical center which provided physician-led medical support 24 h a day, 7 days a week for the entire study period	Usual care	24 months
Kraai et al. [178]	94	83	177	69	37	27	Patients in the telemonitoring group received telemonitoring devices at home consisting of a weighing scale, blood pressure equipment, an ECG-device and a health-monitor. The instruction was to record weight and blood pressure once a day and an ECG in case of starting or up-titration of Beta-blockers. After receiving the data from the above-mentioned devices, the health-monitor generated standard health-related questions regarding the patients’ health status	The ICT-guided-DSM group followed the normal HF-routine of the individual hospitals, like any other HF-patient, without limitations to the visits	9 months
Landolina et al. [181]	101	99	200	68	79	31	ICD-OptiVol	Remote transmission off	16 months
Lüthje et al. [187]	89	87	176	66	77	32	The device determines a representative impedance daily and compares this with a roving reference value. Whenever daily impedance drops below the reference, a cumulative, absolute difference is calculated, and called fluid index	Standard in-office visits were performed every 3 months	15 months
Morgan et al. [199]	824	826	1650	70	86	30	Remote monitoring via an electronic care record form management system	Usual care	34 months
Sardu et al. [227]	89	94	183	72	76	< 35	CRT-D with TM	CRT-D with traditional ambulatory monitoring	12 months
Scherr et al. [228]	54	54	108	66	79	25	Pharmacological treatment with telemedical surveillance for 6 months	Pharmacological treatment	6 months
Soran et al. [312]	160	155	315	76	31	24	Home-based disease management program to monitor and to detect early signs and symptoms of heart failure using telecommunication equipment	Patient 1-on-1 education, an effort to use evidenced-based optimal medical treatment, and a commercially available digital home scale with management by primary physician	6 months
van Veldhuisen [249]	167	168	335	86	86	25	Information available to physicians and patients as an audible alert in case of preset threshold crossings	Information and an alert were not available	15 months
Villani et al. [250]	30	30	60	69	75	31	N/A	N/A	12 months
Villani et al. [251]	40	40	80	72	74	32	The patient front-end operated through a personal digital assistant given to each patient leaving hospital. The cardiologist decided what variables should be followed up (e.g., heart rate, body weight, blood pressure, ECG) and the frequency of monitoring (e.g., daily for blood pressure and body weight, weekly for the ECG) according to the patient’s clinical characteristics	Usual care	1 year
Vuorinen et al. [255]	47	47	94	58	83	27	A patient regularly reported their most important health parameters to the nurse using a mobile phone app. At the beginning of the study, the patients were given a home-care package including a weight scale, a blood pressure meter, a mobile phone, and self-care instructions. The patients were advised to carry out and report the measurements together with the assessment of symptoms once a week	A multidisciplinary care approach including patient guidance and support for self-care has been adopted at the clinic	6 months
Weintraub et al. [256]	95	93	188	69	66	32	Specialized primary and networked care in heart failure disease management program	Disease management program in conjunction with the AHM system	3 months
Zan [263]	N/R	N/R	40	53	N/R	32	Intervention for heart failure self-management over a 90-day study period. Patients were instructed to take their weight, blood pressure, and heart rate measurements each morning	Matched controls	3 months

*ABMMNC* autologous bone marrow mononuclear cell, *ACE* angiotensin-converting enzyme, *AMIO* amiodarone, *ARB* angiotensin II receptor blockers, *BB* beta-blocker, *BID* twice a day, *BiV* biventricular, *BMAC* bone marrow aspirate stem cell concentrate, *BMC* bone marrow cells, *BMMC* bone marrow–derived mast cell, *BMSC* bone marrow stromal cells, *CA* catheter ablation, *CABG* coronary artery bypass graft, *CFAE* complex fractionated atrial electrogram, *CPC* circulating blood, *CPET* cardiopulmonary exercise test, *CR* cardiac rehabilitation, *CRT* cardiac resynchronization therapy, *CRT* cardiac resynchronization therapy, *CRT-D* cardiac resynchronization therapy defibrillator, *CSC* cardiac stem cells, *ET* exercise training, *G-CSF* granulocyte-colony stimulating factor, *HIIT* high-intensity interval training, *ICD* implantable cardioverter defibrillator, *INR* international normalized ratio, *LAPWI* left atrial posterior wall isolation, *LVEF* left ventricular ejection fraction, *MCT* moderate-intensity continuous training, *MDC* multidisciplinary clinics, *MRA* mineralocorticoid receptor antagonists, *PVI* pulmonary vein isolation, *RVP* right ventricular pacing, *STS* structured telephone support, *SVCI* systemic vascular conductance index, *TM* telemonitoring, *VVIR* ventricular rate modulated pacing

### Quality assessment

Overall, risk of bias was classified as relatively low (Table 5). Of the 44 meta-analyses, 11 scored critically low, 15 low, 1 moderate, and 17 high. Almost all meta-analyses registered their protocol before commencement of the review (item 2) and used appropriate meta-analytical methods (item 11). Reviews were mostly downgraded based on the lack of an adequate investigation of publication bias (item 15).

**Table 5:** AMSTAR 2 scores

	Critical domains					Non-critical domains	Judgment
	Item 2^a^	Item 9^b^	Item 11^c^	Item 13^d^	Item 14^e^		
Adamson et al. [266]	●●●	●	●●●	●	●●●	●●	Critically low
Agasthi et al. [267]	●●●	●●●	●●●	●●●	●●●	●●	High
Al-Majed et al. [268]	●●●	●●●	●●●	●	●●●	●●	Low
Alotaibi et al. [269]	●●●	●●●	●●●	●●●	●●●	●●	High
AlTurki et al. [270]	●●●	●●●	●●●	●●●	●●●	●●	High
Benito-González et al. [271]	●●●	●●●	●●●	●●●	●●●	●●	High
Bertaina et al. [272]	●●●	●●●	●●●	●●●	●	●●	Low
Bjarnason-Wehrens et al. [273]	●●●	●●●	●●●	●●●	●●●	●●	High
Bonsu et al. [274]	●●●	●●●	●●●	●●●	●●●	●●	High
Carbo et al. [275]	●●●	●●●	●●●	●●●	●●●	●●	High
De Vecchis et al. [276]	●●●	●●●	●●●	●	●●●	●●	Low
Driscoll et al. [277]	●●●	●●●	●●●	●●●	●●●	●●	High
Emdin et al. [278]	●●●	●●●	●●●	●●●	●	●●	Low
Fisher et al. [279]	●●●	●●●	●●●	●●●	●●●	●●	High
Fisher et al. [280]	●●●	●●●	●●●	●●●	●●●	●●	High
Gandhi et al. [281]	●●●	●●●	●●●	●●●	●●●	●●	High
Halawa et al. [282]	●●●	●●●	●●●	●●●	●●●	●●	High
Hartmann et al. [283]	●●●	●●●	●	●	●●●	●●	Critically low
Hu et al. [284]	●●●	●●●	●●●	●●●	●	●●	Low
Inglis et al. [285]	●●●	●●●	●●●	●	●	●●	Critically low
Inglis et al. [286]	●●●	●●●	●●●	●●●	●●●	●●	High
Japp et al. [287]	●●	●	●●●	●	●	●●	Critically low
Jonkman et al. [288]	●●●	●●●	●●●	●	●	●●	Critically low
Kang et al. [289]	●	●	●●●	●	●	●●	Critically low
Klersy et al. [290]	●	●●●	●●●	●	●●●	●●	Critically low
Komajda et al. [291]	●●●	●●●	●●●	●●●	●	●●	Low
Le et al. [292]	●●●	●	●●●	●	●	●●	Critically low
Ma et al. [293]	●●●	●●●	●●●	●	●●●	●●	Low
Malik et al. [294]	●●●	●●●	●●●	●●●	●●●	●●	High
Moshonas et al. [295]	●●●	●●●	●●●	●●●	●●●	●●	High
Pandor et al. [296]	●●●	●●●	●●●	●●●	●	●●	Low
Shah et al. [297]	●●●	●	●●●	●	●	●●	Critically low
Sulaica et al. [298]	●●	●●●	●●●	●●●	●●●	●●	Moderate
Taylor et al. [299]	●●●	●●●	●●●	●●●	●●●	●●	High
Thomas et al. [300]	●●●	●●●	●●●	●●●	●	●●	Low
Thomsen et al. [301]	●●●	●	●●●	●	●●●	●●	Critically low
Tse et al. [302]	●●●	●●●	●●●	●●●	●●●	●●	High
Tu et al. [303]	●●●	●●●	●●●	●●●	●	●●	Low
Turagam et al. [304]	●●●	●●●	●●●	●●●	●	●●	Low
Uminski et al. [305]	●●●	●●●	●●●	●●●	●	●●	Low
Xiang et al. [306]	●●●	●	●●●	●	●●●	●●	Critically low
Zhang et al. [307]	●●●	●●●	●●●	●	●●●	●●	Low
Zhang et al. [308]	●●●	●●●	●●●	●●●	●	●●	Low
Zhou and Chen [309]	●●●	●●●	●●●	●●●	●	●●	Low

^a^Registered protocol before commencement of the review

^b^Risk of bias from individual studies being included in the review

^c^Appropriateness of meta-analytical method

^d^Consideration of risk of bias when interpreting the results of the review

^e^Assessment of presence and likely impact of publication bias

### Study characteristics

A total of 425,220 patients were included in the 44 meta-analyses and 186 RCTs (Table 4). RCTs included between 16 and 10,917 patients. The mean age of patients ranged from 33 to 96 years. Mean LVEF varied between 17 and 40%. Percentage of male patients ranged from 25 to 100%. Follow-up period ranged widely from 30 days to 10 years. Studies that tried to prevent hospital admissions with cardiac rehabilitation focused on either exercise only or multicomponent cardiac rehabilitation. Care pathways could be divided into either TM, STS, and self-management promotion programs or multidisciplinary clinics. Invasive therapy encompassed catheter ablation (CA), cardiac resynchronization therapy (CRT), mitral valve repair, or stem cell therapy. Medication subtypes were angiotensin-converting enzyme inhibitors (ACE), angiotensin II receptor blockers (ARBs), mineralocorticoid receptor antagonists (MRAs), beta-blockers, statins, anticoagulation, and a miscellaneous subcategory.

### Effect of interventions

#### Primary analysis: meta-analyses

**Fig. 2:**
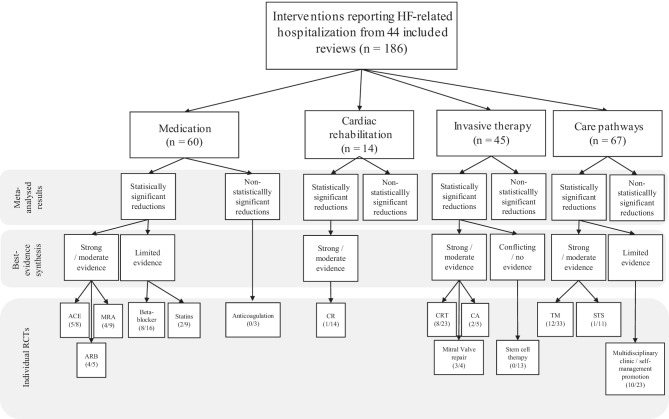
Effects of different interventions on HF-related hospitalization in meta-analyzed and single-study results. ACE, angiotensin-converting enzyme inhibitors; ARB, angiotensin II receptor blockers; MRA, mineralocorticoid receptor antagonists; CR, cardiac rehabilitation; CRT, cardiac resynchronization therapy; CA, catheter ablation; TM, telemonitoring; STS, structured telephone support

Meta-analytic results of the 44 included meta-analyses are demonstrated in Table 6 and Fig. 2. According to our best-evidence synthesis, strong evidence suggests that CA, CR, and TM could prevent heart failure hospitalization. Furthermore, moderate evidence was found for the effectiveness of RAAS inhibitors, and CRT in reducing HF-related hospitalizations, while only limited evidence suggests the beneficial effects of beta-blockers, statins, mitral valve therapy, and multidisciplinary clinics or self-management promotion programs. There is conflicting evidence regarding the effect of cell therapy on HF hospitalization, and no evidence was found that anticoagulation should reduce HF-related hospitalizations.

**Table 6:** Effectiveness of interventions

Author, year	Category	Sig	Conclusion	Statistics
Adamson et al. [266]	Care pathways	√	*Haemodynamic-guided HF management* is superior in reducing long-term HF-hospitalization risk	HR: 0.63 (0.54–0.73)
Alotaibi et al. [269]	Care pathways	√	A significant reduction in HF-hospitalizations in patients undergoing *catheter ablation*	RR: 0.56 (0.44–0.71)
Carbo et al. [275]	Care pathways	√	We found reduction trends in HF-related admissions due to *m-Health*	SMD: − 0.43 (–0.83|–0.02)
Driscoll et al. [277]	Care pathways	√	*Nuse-led titration* may result in a significant reduction in hospital admissions	RR: 0.51 (0.36–0.72)
Gandhi et al. [281]	Care pathways	×	*Multidisciplinary* heart failure clinics failed to show a reduction in HF hospitalization	OR: 0.68
Halawa et al. [282]	Care pathways	×	Usage of *intra-cardiac devices* is not linked to improving rates of HF admission	OR: 1.25 (0.92–1.69)
Inglis et al. [285]	Care pathways	√	Both *STS* and *TM* reduced HF-related hospitalizations	RR: 0.77 (0.68–0.87)^c^
				RR: 0.79 (0.67–0.94)^d^
Inglis et al. [286]	Care pathways	√	*STS* and *TM* improve outcomes for patients with CHF	RR: 0.77 (0.68–0.87)^c^
				RR: 0.79 (0.67– 0.94)^d^
Jonkman et al. [288]	Care pathways	×	No specific program characteristics were consistently associated with better effects of *self-management interventions*	RR: 0.96 (0.92–0.995)
Klersy et al. [290]	Care pathways	√	*TM* was associated with a significantly lower number of hospitalizations for HF	IRR: 0.77 (0.65–0.91)
Pandor et al. [296]	Care pathways	×	There were no major effects on HF-related hospitalization for STS HM (HR: 1.03, 95% CrI: 0.66, 1.54) or TM with medical support during office hours	HR: 1.03, (0.66, 1.54)^c^
				HR: 0.95, (0.70, 1.34)^d^
Thomas et al. [300]	Care pathways	√	*Specialist clinics* for patients with HF can reduce the risk of unplanned admissions	RR: 0.51 (0.41–0.63)
Tse et al. [302]	Care pathways	√	Hospitalization rates can be reduced by remote patient monitoring using either *TM* or *hemodynamic monitoring*	HR: 0.73 (0.65–0.83)^d^
				HR: 0.60 (0.53–0.69)^m^
Uminski et al. [305]	Care pathways	√	A *post-discharge virtual ward* can provide added benefits to usual care to reduce HF-related hospital admissions	RR: 0.61 (0.49–0.76)
Xiang et al. [306]	Care pathways	√	*Telehealth* had a significant overall effect on CHF hospitalization	RR: 0.72 (0.61–0.85)
Bjarnason-Weherens et al. [273]	CR	√	*Exercise-based intervention* reduces the level of hospitalizations due to HF	RR: 0.59 (0.12–2.91)
Taylor et al. [299]	CR	√	*ExCR* did reduce HF-specific hospitalization	RR: 0.59 (0.42–0.84)
Agasthi et al. [267]	Invasive therapy	√	*CA* was associated with significantly lower rate of HF-readmission	RR: 0.58 (0.46–0.81)
Al-Majed et al. [268]	Invasive therapy	√	*CRT* reduces HF-hospitalization in patients	RR: 0.69 (0.58–0.82)
AlTurki et al. [270]	Invasive therapy	√	*RM* showed benefit in reducing HF-related hospitalization when compared to standard of care	RR: 0.95 (0.78–1.16)
Benito-González et al. [271]	Invasive therapy	√	TMVR with *MitraClip*^®^ system was related to a significant reduction in hospitalizations for HF	HR: 0.65 (0.46–0.92)
Bertaina et al. [272]	Invasive therapy	√	*MitraClip* for FMR in patients with LV dysfunction is associated with a considerable reduction of HF-hospitalization	OR: 0.49 (0.24–1.00)
Fisher et al. [279]	Invasive therapy	√	*Cell treatment* is associated with a significant reduction of rehospitalization caused by worsening HF	RR: 0.39 (0.22–0.70)
Fisher et al. [280]	Invasive therapy	×	*Cell therapy* does not appear to reduce the risk of rehospitalization for HF	RR: 0.62 (0.36–1.04)
Ma et al. [293]	Invasive therapy	√	*CA* reduced risks of HF readmission	RR: 0.58 (0.46–0.66)
Malik and Aronow [294]	Invasive therapy	√	*CA* was effective in reducing hospitalization for HF	OR: 0.41 (0.28–0.59)
Moschonas et al. [295]	Invasive therapy	√	In patients randomized to *AFA*, there were significant improvements in unplanned hospitalization rates	RR: 0.58 (0.46–0.73)
Tu et al. [303]	Invasive therapy	√	*CRT* had a marked effect in reducing new hospitalizations for worsening HF	RR: 0.69 (0.60–0.79)
Turagam et al. [304]	Invasive therapy	√	*CA* was associated with reductions in HF hospitalizations	RR: 0.60 (0.39–0.93)
Bonsu et al. [274]	Medication	√	Superiority of lipophilic *statin* treatment in decreasing hospitalization for worsening HF	OR:0. 49 (0.36–0.67)^a^
				OR: 0.94 (0. 86–1.03)^b^
De Vecchis and Ariano [276]	Medication	√	*ARA* use in patients with heart failure was associated with a significant reduction in hospitalization	OR: 0.73 (0.61–0.89)
Emdin et al. [278]	Medication	√	*RAAS* inhibition overall reduces the risk for hospitalization for HF	RR: 0.80, (0.77–0.83)
Gandhi et al. [313]	Medication	√	In patients with acute advanced CHF concomitant *hypertonic saline administration* decreased HF-rehospitalization	RR: 0.50 (0.33–0.76)
Hartmann et al. [283]	Medication	×	*Ivabradine* showed no significant effect for hospitalization due to HF	RR: 0.87 (0.68–1.12)
lTurki al. [284]	Medication	√	The use of *AldoAs* may exert beneficial effects in reducing re-hospitalization for cardiac causes	RR: 0.62 (0.52–0.74)
Japp et al. [287]	Medication	√	*MRAs* did improve hospitalizations	HR: 0.62 (0.47–0.82)
Kang et al. [289]	Medication	√	There was a trend towards reduced HF hospitalization risk with *RAS inhibitors*	RR: 0.91 (0.83–1.01)
Komajda et al. [291]	Medication	√	Disease-modifying *medications* resulted in the progressive improvement in hospitalization outcomes	HR: 0.25 (0.07–0.99)
Le et al. [292]	Medication	√	Significant relative risk reduction of CV hospitalization was observed in those assigned to *AAs*	RR: 0.79 (0.68–0.91)
Shah et al. [297]	Medication	×	Pooled analysis of these trials suggests no consistent benefit of *RAS* inhibition with regard to HF hospitalization	OR: 0.90 (0.80–1.02)
Sulaica et al. [298]	Medication	×	No difference was noted between the *anticoagulation* and placebo group in regard to hospitalization for HF	OR: 0.97 (0.80–1.18)
Thomsen et al. [301]	Medication	√	Drugs targeting the *renin–angiotensin–aldosterone system*, *beta-blockers*, *digoxin*, and *CRT* significantly reduced the risk of HF hospitalization	RR: 0.71 (0.65–0.78)^e^
				RR: 0.63 (0.44–0.91)^f^
				RR: 0.76 (0.64–0.90)^g^
				RR: 0.78 (0.73–0.82)^h^
				RR: 0.40 (0.20–0.78)^i^
				RR: 0.87 (0.68–1.11)^j^
				RR: 0.64 (0.57–0.71)^k^
				RR 1.34 (1.04–1.73)^l^
Zhang et al. [307]	Medication	√	The beneficial effects of *TMZ* have been demonstrated by the decrease of hospitalization	RR: 0.43 (0.21–0.91)
Zhang et al. [308]	Medication	×	Our meta-analysis suggests that *liraglutide* treatment has no important influence on hospitalization for HF	RR: 1.18 (0.88–1.58)
Zhou and Chen [309]	Medication	√	*TMZ* treatment in CHF patients may reduce hospitalization for cardiac causes	RR: 0.43 (0.21–0.91)

*HF* heart failure, *CA* catheter ablation, *CR* cardiac rehabilitation, *CRT* cardiac resynchronization therapy, *STS* structured telephone support, *UF* ultrafiltration, *TMZ* Trimetazidine, *TM* telemonitoring

^a ^Lipostatin

^b^ Rosuvastatin

^c ^Structured telephone support

^d ^Telemonitoring

^e ^ACE

^f ^ARB

^g ^ARA

^h ^Beta-blocker

^i ^Digoxin

^j ^Ivabradine

^k ^CRT

^l ^ICD

^m ^Hemodynamic monitoring

#### Secondary analysis: extracted RCTs

In order to prevent bias as a result of duplicated data, all unique RCTs (*N* = 186) were extracted in a secondary analysis from the meta-analyses and compared to the results from our primary analysis.

#### Cardiac rehabilitation

**Fig. 3:**
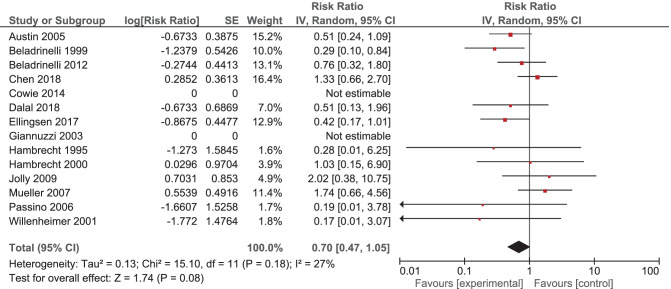
Forest plot of RR for HF-related hospitalization between cardiac rehabilitation and control. Random effects model

A total of 14 studies examined the effects of cardiac rehabilitation. Of these individual studies, 1 reported a significant effect. When examined in a meta-analysis, a significant positive effect of cardiac rehabilitation was found (RR: 0.66, 95% CI: 0.44 | 0.97) (Fig. 3). This is in accordance with the general findings reported by the studied meta-analyses. Upon visual inspection, the funnel plots suggest no publication bias (Fig. 7).

#### Invasive therapy

**Fig. 4:**
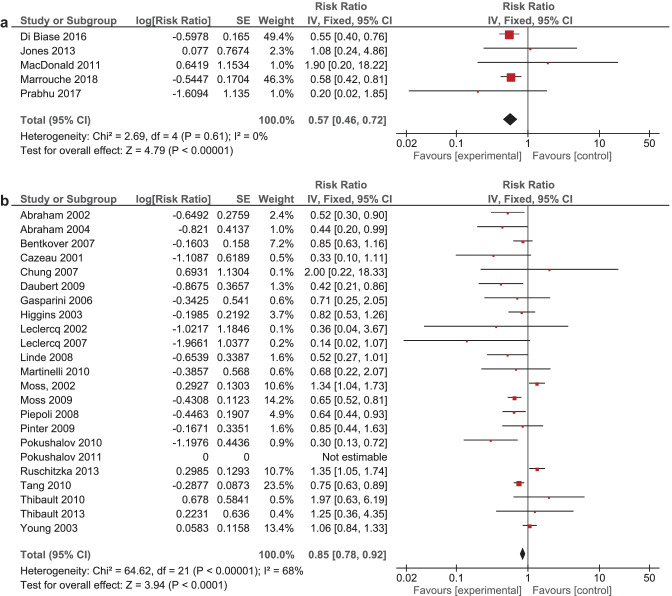

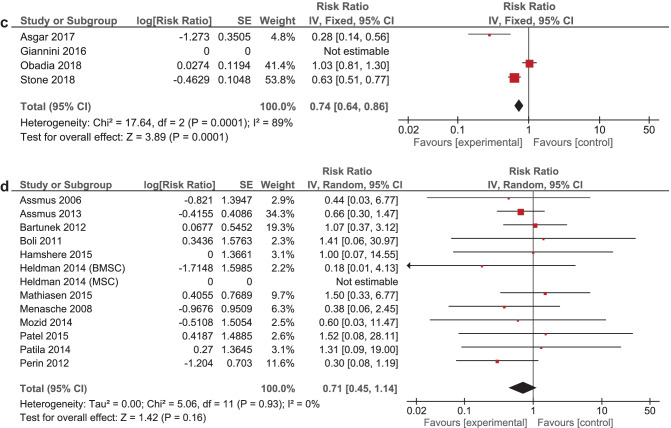
(**A**–**D**) Forest plots of RR for HF-related hospitalization between (**A**) catheter ablation, (**B**) cardiac resynchronization therapy, (**C**) mitral valve therapy, and (**D**) stem cell therapy, and control. Fixed effects model

There were 5 studies examining the effect of CA. Of these studies, 2 studies reported a significant effect. A positive effect of CA on HF-related hospitalization was found in our meta-analyses (RR: 0.57, 95% CI: 0.46 | 0.72) (Fig. 4). This is consistent with the general findings reported by the studied meta-analyses.

A total of 23 studies examined CRT to prevent HF-related hospitalization. Of these, 8 studies found a positive effect. Our meta-analysis suggested a positive effect of CRT (RR: 0.85, 95% CI: 0.78 | 0.92). This is in line with the general findings reported by the studied meta-analyses.

Of the 4 studies that examined mitral valve repair, 3 reported an effective reduction in HF-related hospitalization. Our meta-analyses suggested a positive effect (RR: 0.74, 95% CI: 0.64 | 0.86), which is in agreement with the general findings reported by the studied meta-analyses.

Stem cell therapy was in 0 of the 13 studies related to reduced HF-related hospitalization, which is in line with our meta-analyzed result (RR: 0.71, 95% CI: 0.45 | 1.14) and the conflicting evidence suggested by the studied meta-analyses.

The funnel plots indicate no, or only minimal publication bias (Fig. 7).

#### Medication

**Fig. 5:**
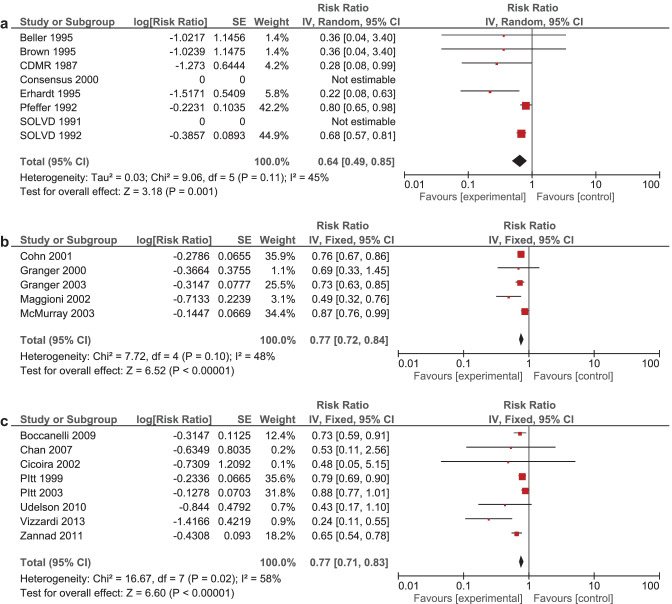

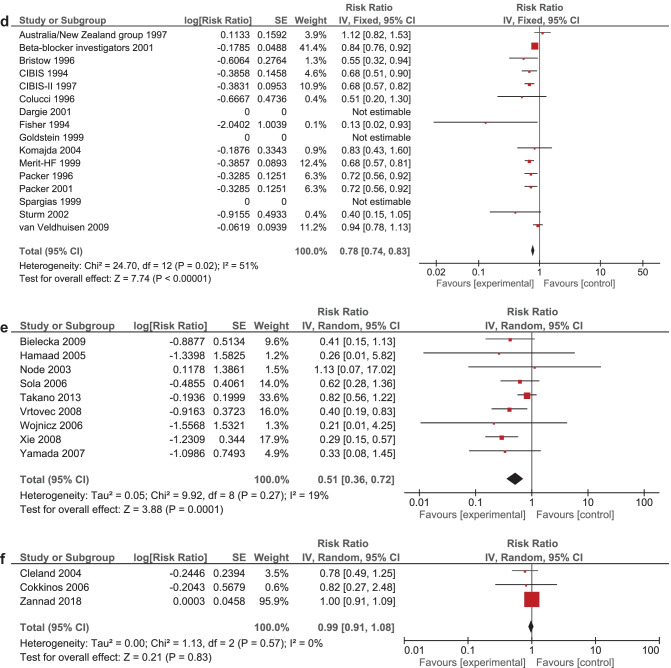
(**A**–**F**) Forest plots of RR for HF-related hospitalization between (**A**) angiotensin-converting enzyme inhibitors, (**B**) angiotensin II receptor blockers, (**C**) mineralocorticoid receptor antagonists, (**D**) beta-blockers, (**E**) statins, and (**F**) anticoagulation, and control. Fixed effects model

ACE inhibitors (5/18 studies; RR: 0.64, 95% CI: 0.49 | 0.85), MRAs (4/9 studies; RR: 0.77, 95% CI: 0.71 | 0.83), ARBs (4/5 studies; RR: 0.77, 95% CI: 0.72 | 0.84), beta-blockers (8/16 studies; RR: 0.78, 95% CI: 0.74 | 0.83), and statins (2/9 studies; RR: 0.51, 95% CI: 0.36 | 0.72) showed a significant effect of reduced hospitalizations in our meta-analyses (Fig. 5). This is in line with the general findings reported by the studied meta-analyses.

Anticoagulation (RR: 0.99, 95% CI: 0.91 | 1.08) was in none of the studies (0/3) able to reduce HF-related hospitalizations. This absence of an effect was also reported by the studied meta-analyses.

The asymmetry in the medication funnel plots suggests some publication bias towards significant effectiveness of medication in reducing HF-related hospitalizations (Fig. 7).

#### Care pathways

**Fig. 6:**
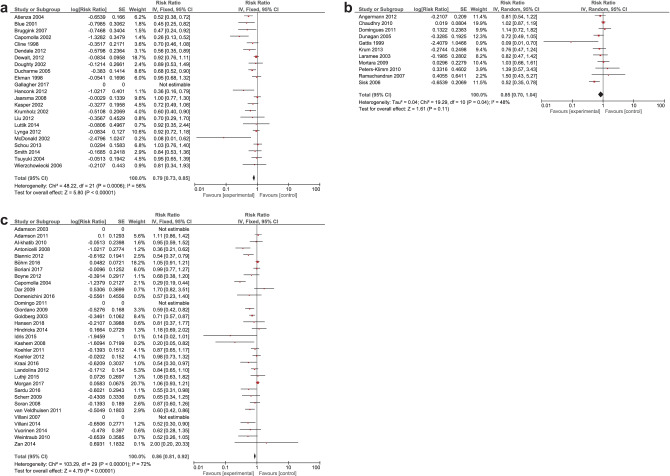
(**A**–**C**) Forest plot of RR for HF-related hospitalization between (**A**) multidisciplinary clinics or self-management promotion programs, (**B**) structured telephone support, and (**C**) telemonitoring, and control. Fixed effects model

**Fig. 7:**
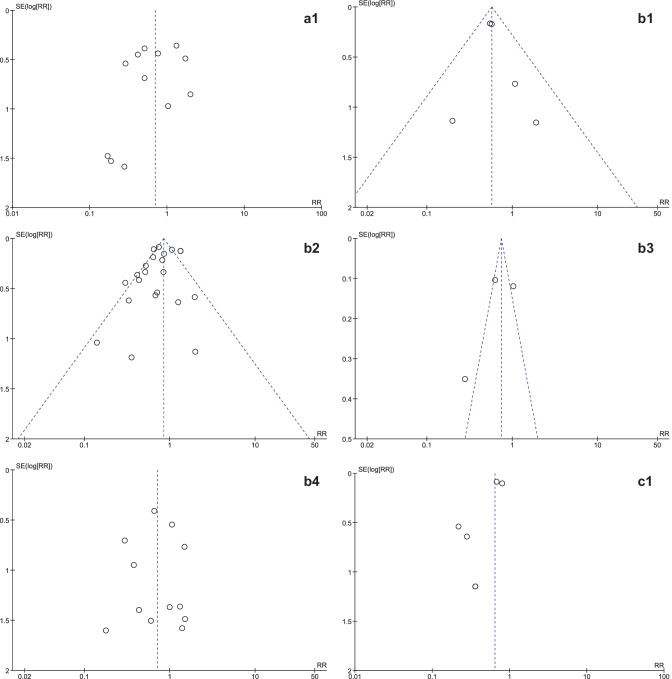

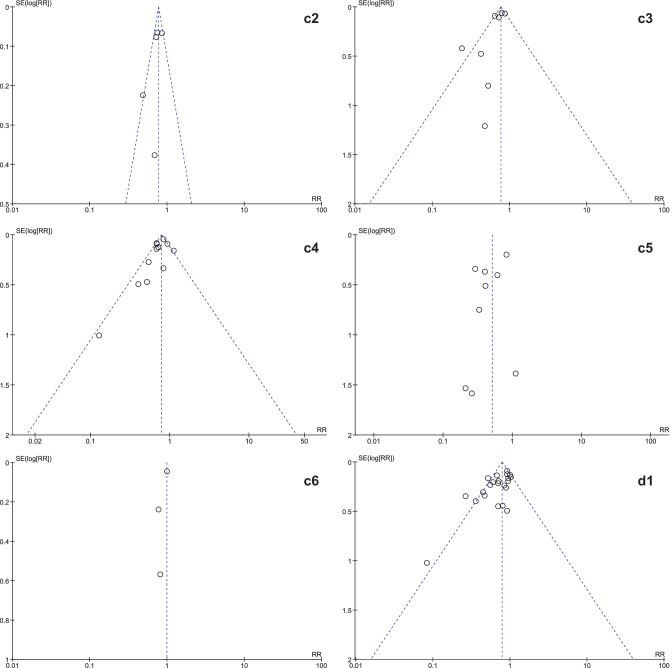

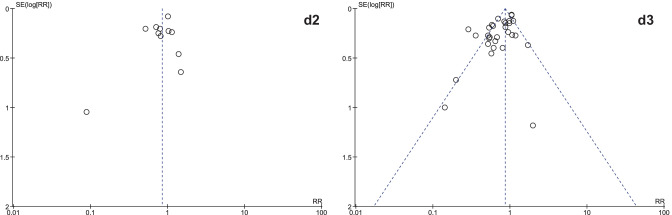
(**A**–**D**) Funnel plots of the effects of (**A**) cardiac rehabilitation, (**B**) telemonitoring, (**C**) medication, and (**D**) invasive therapy

Multidisciplinary clinics or self-management promotion programs (10/23 studies; RR: 0.79, 95% CI: 0.73 | 0.85) and TM (12/33 studies; RR: 0.86, 95% CI: 0.81 | 0.92) were related to less HF-related hospitalizations (Fig. 6). This is in agreement with findings reported by the studied meta-analyses. STS (1/11 studies; RR: 0.85, 95% CI: 0.85 | 1.04) was not related to reductions in HF-related hospitalizations. This is in contrast to findings from the meta-analyses. Visual inspection of the funnel plots did not suggest publication bias (Fig. 7).

## Discussion

Heart failure is a major health concern, with the highest readmission rates among all diseases [8–11]. Yet, up to 40% of hospitalizations could be classified as preventable [36–40]. This umbrella review therefore aimed to systematically review all published meta-analyses conducted in the past 10 years that examined the incremental benefit of interventions in addition to standard care, in reducing HF-related (re)hospitalization, in order to provide a comprehensive overview of different levels of evidence with regard to the different interventions that aim to reduce HF-related (re)hospitalization.

Even though previous studies did examine the effectiveness of interventions in treatment for heart failure in general, this umbrella review highlights different levels of evidence regarding the effectiveness of several interventions in *reducing HF-related hospitalization*. All different categories of interventions (i.e., cardiac rehabilitation, invasive treatment, medication, and care pathways) entail interventions that prove able to statistically significantly reduce HF-related hospitalizations. Strong or at least moderate evidence was found for the beneficial effects of CA, CRT, ACE inhibitors, MRAs, ARBs, CR, TM, and STS. Limited evidence was found for the ability of beta-blockers,, statins, mitral valve repair, and multidisciplinary clinics or self-management promotion programs to reduce hospitalization rates. Conflicting or no evidence was found for the effects of anticoagulation and stem cell therapy.

The findings of this umbrella review were generally supported by the American Heart Association and European Society of Cardiology heart failure guidelines [46, 64]. Yet, evidence for effectiveness was still lacking for several interventions in these guidelines. A couple of interventions proposed in the guidelines had low levels of evidence, as they were only supported by a single randomized clinical trial. Although these guidelines do not solely focus on the prevention of (re)hospitalization, this umbrella review now provides additional evidence for the effectiveness of ARBs (e.g., Valsartan) and telemonitoring as effective in the prevention of (re)hospitalization in heart failure. Therefore, the results of this review may be used in addition in clinical practice, as well as by policymakers, as a guideline in deciding what treatment option might help prevent hospitalization in at risk heart failure patients.

Effectiveness of reported interventions was measured in terms of a reduced risk for heart failure related hospitalizations. However, it would be naïve to suggest that this equals the clinical, genuine effect of treatment. Non-effectiveness of treatment could also be related to non-adherence or non-acceptance of the intervention by the patient, since it is estimated that non-adherence ranges between 30 and 50% in patients with chronic illnesses [65]. And non-adherence not only holds for medication, yet also for cardiac rehabilitation [66, 67] and telemonitoring [68, 69]. It has been shown that worsening of HF is often related to non-adherence of patients [70] and is in fact associated with 10% of hospitalizations [65, 71] and a 10% increased risk of readmission [72]. The other way around, reductions in non-adherence are found to result in less hospital admissions [73].

Differences in non-adherence to different forms of interventions were also found. For example, patients are found to be more adherent to ACE-inhibitors (77.8%) as compared to beta-blockers (69.8%) [74]. These differences could be explained by cognitions of patients regarding the efficacy of the intervention and the usability of the intervention [75]. Moreover, low health literacy or simply a lack of knowledge about the syndrome could also contribute to non-adherence [76–78]. In clinical practice, one should therefore educate patients about the importance of disease management with medication, invasive therapy, cardiac rehabilitation, and care pathways [65, 79].

Moreover, when implementing interventions in practice, one should not only focus on effectiveness, yet also incorporate, for example, the costs of the intervention. Especially, since HF is the most costly condition in western countries, with at least twice the costs of the estimated consumption of healthcare in the general population in a year [32, 33, 80], mainly due to HF-related hospitalization [28, 29]. Research has shown that, in terms of cost-effectiveness, medication treatment with beta-blockers, ARBs, or ACE inhibitors could be preferred over more cost expensive therapies as device therapy with CRT [81, 82]. More specifically, with regard to specific forms of medication, ivabradine seems a cost-effective treatment option, while this does not hold for valsartan [82]. In addition, general HF treatment combined with telemonitoring has been found to be between 27 and 52% more cost-efficient than usual care alone [83, 84]. Furthermore, telemonitoring seems not only cost-efficient; but nowadays, with the pandemic consequences of COVID-19 it seems more desired than ever [85]. The pandemic served as a catalyst, as both healthcare professionals as patients wanted optimal care in a time of reduced ambulatory outpatient clinics, with being compliant to social distancing [84]. Our review shows, in addition, that, even though the terms are interchangeably used to both describe some form of “remote care,” telemonitoring and structured telephone support have different levels of effectiveness with regard to prevention of heart failure related (re)hospitalizations, which should be accounted for in clinical practice.

In this umbrella review, we only aimed to provide an overview of effective treatment options for prevention of heart failure (re)hospitalization. Consequently, no conclusions could be drawn regarding the hierarchy of effectiveness based upon this review. In future research, it should be examined what factors contribute to effectiveness of interventions, as our study only showed that particular interventions could reduce heart failure hospitalizations, but not *why* per se. Research should focus on the effective mechanisms of care pathway programs, for example, or on determinants of successful implementations of interventions for heart failure.

The aim of our review was to assess interventions which are currently used in clinical practice and examined in large populations. Our results are based upon meta-analyses performed within the past 10 years. Yet, most recent innovative treatment options are probably underrepresented. For example, no study examined the effects of SGLT-II inhibitors, while the European Society of Cardiology stated that SGLT-II inhibitors could be preferred in heart failure patients [86]. Future studies should examine whether the use of SGLT-II inhibitors could show effective in reducing hospitalization. Moreover, as the aim of our review was to assess interventions which are currently used in clinical practice and examined in large populations, we expected to find multiple meta-analyses examining the same interventions. A large amount of overlap in RCTs in included meta-analyses was found. This stresses the importance of registration of protocols and knowing whether the intended research subject has a significantly different research objective than existing, or outdated reviews [62].

To conclude, this umbrella review highlights different levels of evidence regarding the effectiveness of several interventions in reducing HF-related hospitalization in HFrEF patients. It provides an overview of all, known, meta-analyses conducted in the past 10 years that examined interventions to prevent heart failure related hospitalizations. All different categories of interventions entail interventions that prove able to statistically significantly reduce HF-related hospitalizations. Most evidence was found for the beneficial effects of angiotensin-converting enzyme inhibitors (ACE), mineralocorticoid receptor antagonists (MRAs), angiotensin II receptor blockers (ARBs), cardiac rehabilitation, and telemonitoring. The results of this review may be used in clinical practice, as well as by policymakers, to guide treatment for heart failure patients at risk of hospitalization.
